# Investigating Metabolically Altered Pathways in Small Cell Lung Cancer: From RNA Sequencing Analysis to Seahorse-Based Functional Validation

**DOI:** 10.3390/mps9020046

**Published:** 2026-03-10

**Authors:** Subhadeep Das, Sagar M. Utturkar, Roshnee Bose, Elizabeth J. Tran

**Affiliations:** 1 Department of Biotechnology, School of Life Science & Biotechnology, Adamas University, Kolkata 700126, India; 2 Purdue Institute for Cancer Research, Purdue University, Hansen Life Sciences Research Building, Room 141, 201 S. University Street, West Lafayette, IN 47907-2064, USA; sutturka@purdue.edu; 3 Department of Comparative Pathobiology, Purdue University, IN 47907, USA; roshneebose2020@gmail.com; 4 Department of Biochemistry, Purdue University, BCHM A343, 175 S. University Street, West Lafayette, IN 47907-2063, USA

**Keywords:** DEAD-box helicase 5 (DDX5), small cell lung cancer, RNA sequencing, Seahorse XF Cell Mito Stress Test, mitochondrial dysfunction

## Abstract

Small cell lung cancer (SCLC) is an aggressive malignancy characterized by rapid progression, early metastasis, and high relapse rates due to acquired chemoresistance. The human DEAD-box RNA helicase DDX5 is overexpressed in SCLC and has recently gained attention as a viable therapeutic target. Supinoxin (RX-5902), a selective small-molecule inhibitor of DDX5, exhibits strong anti-tumor activity. Recent evidence suggests that its cytotoxic effects are mediated through the disruption of mitochondrial respiration. In this study, transcriptomic profiling via RNA sequencing (RNA-seq) revealed significant downregulation of genes involved in cellular respiration following Supinoxin treatment and DDX5 knockdown in chemoresistant H69AR cells. To functionally validate these findings, we employed the Seahorse XF Cell Mito Stress Test, which measures key parameters of mitochondrial bioenergetics through oxygen consumption rate (OCR) analysis. Supinoxin-treated cells exhibited marked mitochondrial dysfunction, supporting the hypothesis that DDX5 inhibition disrupts cellular energy metabolism. These findings illuminate a previously underappreciated role of DDX5 in mitochondrial regulation and offer mechanistic insights into Supinoxin’s cytotoxic effects, underscoring its potential as a targeted therapy in SCLC.

## 1. Introduction

Lung cancer emerged as the predominant cause of cancer morbidity and mortality in 2022, with nearly 2.5 million new cases and over 1.8 million deaths globally [1]. It accounted for approximately 12.4% of all diagnosed cancers and 18.7% of cancer-related deaths, as reported by GLOBOCAN 2022. In 2022, it was estimated that there were 1,572,045 new cases of lung cancer globally among males, with 180,063 (11.5%) classified as small-cell carcinoma (small cell lung cancer, SCLC). Among females, there were 908,630 new cases, with 87,902 (9.7%) identified as small-cell carcinoma worldwide (small cell lung cancer, SCLC) [2]. Small cell lung cancer (SCLC) is currently considered a recalcitrant malignancy that accounts for approximately 15% of all lung cancer cases, exhibiting a five-year survival rate of under 7% [3,4]. The current standard of care involves a combination therapy comprising etoposide and a platinum-based chemotherapeutic agent, along with immune checkpoint inhibition using anti-programmed death-ligand 1 (anti-PD-L1) antibodies [5]. Most patients receiving this regimen eventually develop chemoresistance, resulting in rapid disease progression and a poor long-term prognosis, with only a 10–20% survival rate at two years [6]. There is a dire need to develop alternative treatment strategies for this malignancy.

The human DEAD-box helicase 5 (DDX5) is an RNA helicase that is involved in various key aspects of RNA metabolism. Dysregulation of DDX5 has been implicated in several cancer types, including colon [7], prostate [8], breast [9], leukemia [10], and lung cancers [11,12], where it contributes to tumor growth both [10] in vitro and in vivo. In prostate cancer, its overexpression enhances mTOR pathway activation, promoting tumor growth [13]. In colorectal cancer, DDX5 cooperates with the Wnt/β-catenin signaling pathway to transcriptionally activate oncogenic targets like Carboxy terminus of Hsc70-interacting Protein (CHIP), thereby facilitating increased cell proliferation and migration [14]. It also plays a nuanced role in the p53 pathway by selectively enhancing p53-mediated transcription of cell cycle arrest genes while modulating apoptotic responses, balancing cell survival and programmed cell death [15].

Previous studies indicate that the RNA helicase DDX5 is essential for the invasive growth of SCLC. Knockdown of DDX5 leads to a significant disruption in mitochondrial respiration and cell growth [11]. This effect is linked to reduced TCA cycle activity, as evidenced by lower intracellular succinate levels, which typically supply electrons to mitochondrial Complex II via succinate oxidation [11]. Furthermore, knockdown of DDX5 also downregulates nuclear-encoded mitochondrial genes in SCLC cells, thereby reducing the capacity of these cancer cells to produce the necessary energy for vital cellular functions [11]. Nuclear DNA-encoded mitochondrial genes are essential for maintaining mitochondrial homeostasis by influencing the expression of mitochondria-related genes in cancer cells [16,17,18,19].

Supinoxin (RX-5902) [20], developed by Rexahn Pharmaceuticals (Rockville, MD, USA), is a small-molecule inhibitor targeting phosphorylated DDX5 (pDDX5) that has demonstrated potent anti-cancer activity across a range of tumor models in both in vitro and in vivo [21]. Initially, it was believed that Supinoxin exerts its anti-cancer effects by disrupting the interaction between phosphorylated DDX5 (pDDX5) and β-catenin, thereby inhibiting the nuclear translocation of β-catenin in breast cancer cells (MDA-MB-231) [9,22]. Consequently, it would suppress the Wnt/β-catenin signaling pathway that is often hyperactivated in cancers [23]. However, studies conducted in our lab have demonstrated that Supinoxin does not operate via the β-catenin pathway in either SCLC or MDA-MB-231 cells utilized in earlier studies [12]. Rather, treatment of Supinoxin leads to the inhibition of genes linked to oxidative phosphorylation, resulting in compromised mitochondrial function in H69AR SCLC cell lines [12]. This article details a workflow that combines RNA-seq-based pathway investigation [24,25] with Seahorse extracellular flux analysis [26,27] to detect and functionally validate metabolically altered pathways in small cell lung cancer upon Supinoxin treatment or DDX5 knockdown.

Briefly, using RNA-seq, we studied the differential expression of genes (DEGs) in Supinoxin-treated and untreated chemoresistant H69AR cells upon DDX5 knockdown (DDX5KD). 435 transcripts were identified to be downregulated in DDX5KD H69AR cells post Supinoxin treatment. The most statistically significant downregulated genes were involved in mitochondrial respiration, particularly oxidative phosphorylation [12]. To further investigate the impact of Supinoxin, we utilized the Seahorse XF Cell Mito Stress Test, which quantitatively measures key parameters of mitochondrial respiration. This analysis confirmed that Supinoxin disrupts mitochondrial bioenergetics in H69AR cells, supporting the transcriptional evidence of impaired oxidative phosphorylation [12]. This article integrates transcriptomic and metabolic analyses, providing a framework for exploring metabolic vulnerabilities in cancer cells, with potential for wider applications in cancer metabolism and therapeutic assessment. 

For complete details on the use and execution of these methods, please refer to [12].

## 2. Materials and Methods

### 2.1. Determination of Altered Pathways by DDX5 Knockdown (DDX5KD) or Supinoxin Treatment Through RNA-Seq Data Analysis

The chemicals, software, and deposited data used are listed in Table 1.

#### 2.1.1. Preparation

Create a working directory to perform analysis and load the required bioinformatics packages. Detailed prerequisites (Hardware, Bioinformatics software and R-packages) are included in Supplementary Information (Supplement S1).

Note: with the special symbol ~ in this command, we create a working directory in the “/home/” directory of the user account. Please update this location as appropriate and ensure to update paths in subsequent commands to reflect the location on a specific computer.

Download reference data. Raw FASTQ files were obtained from GSE255741 (Supinoxin) and GSE142024 (DDX5). Reference human genome sequence (hg38) in FASTA format and annotation in GTF format were downloaded from Ensembl genome browser (https://www.ensembl.org/). BED format gene annotations for Human (hg38_GENCODE_V47.bed.gz) were downloaded from (https://sourceforge.net/projects/rseqc/files/BED/Human_Homo_sapiens/ (accessed on 2 March 2026)).

Note: The RNAseq data used in the current analysis were published earlier [11,12]. While earlier publications included key RNAseq results (differential-expression, top-enriched pathways) and brief methods, an end-to-end workflow to analyze RNAseq data was not included. The current publication includes a step-by-step workflow with detailed instructions and working code to reproduce the results. This end-to-end RNAseq workflow will serve as a primer to perform standard RNAseq analysis on other relevant datasets.

Note: We have included a specific version of the human genome (release 114) in our download link above to ensure reproducibility. The genome assembly and annotations are frequently updated, so it is recommended to use the latest version of the assembly and annotations. The underlying biology and pathways should not change but results for individual genes, counts, and significance may alter with updates.

#### 2.1.2. Quality Control of FASTQ Data

**Figure 1:**
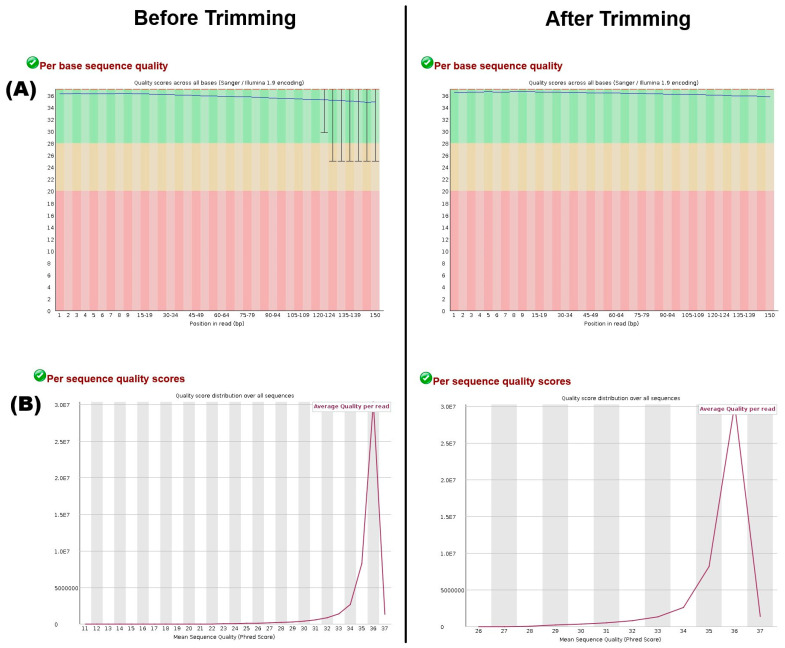
Data overview before and after quality control. (**A**) The per-base sequence quality figure shows how this varies across each read position in a FASTQ file. Per-base sequence quality shows how this varies across each position of the reads in a FASTQ file, using a graph where the *x*-axis is read position, and the *y*-axis is the quality score. The green background indicates high quality, orange indicates reasonable quality, and red indicates poor quality. Key metrics shown are the median (red line), the mean (blue line) and whiskers extend to the 10th and 90th percentiles, showing the spread of quality scores. Note the difference between before and after trimming graphs, where low-quality bases (PHRED score < 30) are removed after trimming, and quality spread is within the green background only. The per-sequence quality score plot shows the distribution of the average quality score across all sequences in a FASTQ file. The *x*-axis represents the average quality score of a read, and the *y*-axis represents the number of sequences that have that average quality score. (**B**) Per sequence quality score plot shows the distribution of the average quality score across all sequences in a FASTQ file.

- Quality assessment for each data was performed using FastQC tool [28].
- Quality-based trimming (removal of adapters, low-quality bases and short sequences) was performed with fastp [29]. After trimming, re-assessment of trimmed data was performed using FastQC to ensure optimal data quality.
- An example of the output from the FastQC tool (before and after quality trimming) is shown in Figure 1.

Note the minimum quality score (*x*-axis) in the before-trimming graph is 11, while the after-trimming graph is 26, denoting that only high-quality bases were retained after trimming. Most sequences have a high quality above 30.

#### 2.1.3. Indexing the Reference Genome

- Indexing of the reference genome (structured representation of a genome to enable faster and more efficient searching and alignment of DNA sequences) was performed through the STAR aligner [30].

#### 2.1.4. Alignment with the Reference Genome

- Quality-trimmed reads were mapped to the reference genome using the STAR aligner. The alignment summary file was inspected to ensure a suitable percentage of reads are mapped to the reference genome. The aligned data (BAM format) were generated for each sample.

Note: outFilterIntronMotifs RemoveNoncanonical option in STAR aligner is used to remove non-canonical splice junctions from the output. Non-canonical splice junctions were filtered out to reduce alignment artifacts and improve the robustness of gene-level quantification. Rare canonical introns (GC–AG, AT–AC) were retained. While noncanonical splicing events may be biologically relevant in cancer, they are often associated with splicing errors and can introduce noise in the data. Noncanonical splicing events are typically rare and require junction-level validation. This was beyond the scope of this study and filtering was appropriate for our (gene-level) analysis goals.

Note: twopassMode Basic option in STAR aligner is used to perform a two-pass alignment. In the first pass, STAR identifies splice junctions from the initial alignment. In the second pass, STAR re-aligns the reads to the augmented junction set and, in turn, improves sensitivity for novel splice junctions and enhances alignment accuracy.

Note: The BAM file is a compressed, binary version of a SAM (Sequence Alignment MAP) file. Although BAM file contents cannot be directly seen/edited, it is preferred for compact size and efficient storage, making it the standard format for alignment data.

Note: Most bioinformatics tools are designed to work directly with BAM-formatted files. Most tools expect BAM files sorted by reference genome coordinates, while a small number of tools may need BAM files sorted by read names. BAM files sorted by read names or coordinates can be easily generated using samtools [31].

Acceptance thresholds for key quality metrics and mapping parameters, along with recommendations for corrective actions when these criteria are not met, are provided in Table 2.

#### 2.1.5. Infer Data-Strandedness

- Determining if data is stranded or un-stranded (dependent on library preparation and sequencing protocols) is crucial for proper analysis and interpretation of RNA-seq data. Stranded protocols preserve the directionality of the transcripts, while non-stranded protocols do not.

Note: infer_experiment.py performs random sampling of mapped reads and counts the sense vs. antisense strand reads that overlap with annotated genes. The output provides the fraction of reads assigned to each strand, which helps to determine the strandedness of the data. We used uniquely mapped reads (mapping quality score of 255) for this analysis and removed bias towards repetitive regions and multi-mapped reads.

Note: The specific parameter ‘-s 1000000’ was used to sample 1 million reads for the inference. This is a common practice to sample noise in low-depth sequencing data and avoid unreliable strandedness estimates.

Note: The chromosome name prefix should be exactly the same as that in the reference genome and the BED file used to determine strandedness. For example, a chromosome named “chr1” vs. “1” in reference and BED file or vice versa is the most common mistake at this step.

- Data strandedness for each sample was determined using the ‘infer_experiment.py’ script from the RSeQC package [32]. The output denotes the fraction of reads assigned to each biological sequence orientation (5′-3′—forward or 3′-5′ reverse). Generally, for un-stranded libraries, fractions of reads assigned to each orientation are roughly equal (50:50) while for stranded libraries a definitive bias is observed towards one orientation (80:20, 20:80 or similar).

#### 2.1.6. Quantification to Generate Counts Matrix

- The count matrix in RNA-seq summarizes the expression level of genes in each sample. It is generated by counting the number of reads aligned to each gene.
- A ‘featureCounts’ program from the subread package [33] was employed to count the reads assigned to genes in each sample. Genome annotation (GTF format), aligned reads (BAM format) and inferred strand information were provided as input and the program counted the reads assigned to each gene within each sample. In the featureCounts’ output (TAB-delimited file), column1 and column 7 corresponding to “Gene ID” and “assigned read counts”, respectively, were extracted using the cut command.
- Lastly, a custom script was employed to generate a combined counts matrix where each row represented a gene, and each column represented a sample. The values in the matrix denote the number of reads mapped to each gene within each sample.

Note: Please inspect and review the ‘combined_counts.tsv’ file contents to ensure that it has appropriate sample names. Users can calculate the SUM across columns to determine the number of reads mapped within genes.

- Transcripts Per Million (TPM) are normalized counts in RNA-seq data. TPM represents the relative abundance of transcripts, essentially indicating the number of reads detected for a gene if sequenced to one million reads. TPM normalizes for both sequencing depth and transcript length, making it useful for comparing gene expression across different samples.

The TPMCalculator program [34] was employed to generate the TPM counts. TPMCalculator adds suffixes as “#1”, “#2”, …, “#N” when the same gene ID is denoted at different locations in an annotation (GTF) file. For the sake of simplicity, we kept the gene location with maximum assigned counts so that the maximum assigned counts for a specific gene (i.e., dominant (highly expressed) transcript) was retained. This approach prioritized the dominant expressed locus for the specific gene while reducing the noise from low-expression transcripts or ambiguity of annotated gene models. This approach avoided artificial inflation of counts that may occur if counts are summed across multiple loci. This strategy is opted for in the context of the current goal of gene-level differential expression analysis. For other goals, such as isoform-specific analyses, the current strategy may not be appropriate, and it is recommended to retain and review the counts for duplicated genes.

**Figure 2:**
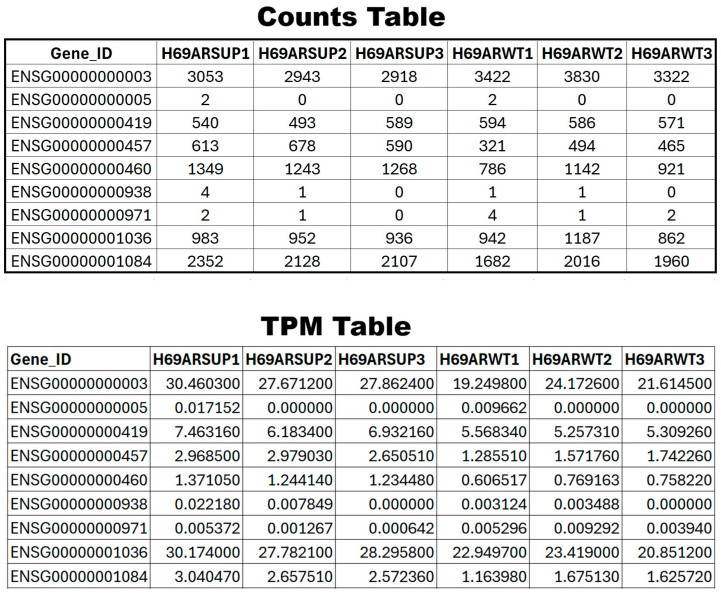
Expected outcome for the quantification step in the RNA-seq pipeline. An overview of expected counts and TPM table is provided, where rows denote the genes, columns denote the replicates/samples, and values denote the respective count and TOM value.

Note: The user should inspect the contents (sample name, gene names, etc.) within the “TPM_final” object to ensure its accuracy before proceeding to the next step. Example output for counts and TPM matrices is shown in Figure 2. In Figure 2, columns denote individual replicates in each group, while rows denote the individual gene. This table typically has counts over thousands of genes across all the replicates probed in specific experiments.

**Figure 3:**
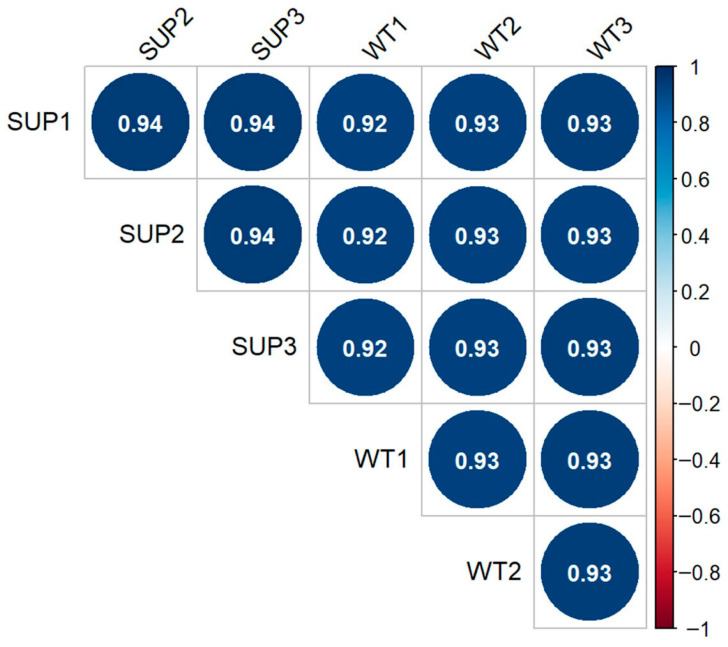
Correlation plot for replicates/samples. The correlation plot shows the Spearman correlation between samples determined using the counts data. In general, high correlation (>0.9) was observed among all samples.

- Next steps determined the correlation between replicates. The ENCODE recommended eplicates concordance is Spearman correlation of >0.9 between isogenic replicates and >0.8 between anisogenic replicates (i.e., replicates from different donors) (Figure 3). We determined Spearman’s correlation among replicates in Supinoxon data and observed high concordance (>0.9) among replicates.
- Recommendations for low correlation among replicates:

Correlation thresholds serve as a screening tool and not an automatic exclusion criterion. Low correlation may represent a true biological variation, and it is important to evaluate various technical aspects. For example, one replicate showing low correlation compared to all others (single outlier) may denote sample-specific technical issues, and the concerned sample should be assessed in terms of sequencing-depth, quality matrices (base-quality, read-length), mapping rate, duplication rate, etc. Technical failures like low-depth sequencing, elevated duplication or modest deviation from quality matrices may be addressed by additional sequencing. On the contrary, technical issues such as significantly low mapping rates (<60%), library size <2× cohort median, duplication rate >2× cohort median, inconsistent strandedness indicate severe failures and may need assessment for experimental anomalies (e.g., RNA degradation, library preparation artifacts or contamination) and may flag the sample for exclusion. If sequencing was performed in batches, then assessment of batch-effect and batch-correction may be necessary as described elsewhere [35]. The ENCODE recommendation is three replicates for each biological condition [36].

#### 2.1.7. Differential Expression (DE) Analysis

- DE analysis identified genes with significant changes in expression levels between two or more conditions. The analysis involved statistical tests to determine if observed differences in gene expression were likely due to biological factors rather than random noise.
- DE analysis between (DDX5-knockdown and control) and (Supinoxin treated and control) was performed using edgeR R-package [37]. A complete script for DE analysis is available on GitHub (https://github.com/sagarutturkar/RNAseq.Seahorse.Validation.TranLab2025) (accessed on 2 March 2026) and on Zenodo (DOI: https://doi.org/10.5281/zenodo.18638583).

Note: Please note that the DE script expects this order: countfile, control_name, treatment_name, number_of_control_replicates, number_of_treatment_replicates, path_for_annotation_file. If control and treatment are specified in the wrong order, the resulting “logFC” will be incorrect.

Note: Use should ensure that the control and treatment names match as in the count matrix. If the count matrix has samples as (control1, control2, control3, treat1, treat2and treat3, for example), the corresponding command to run DE scripts should be “system(“RScript DE_edger.R counts.TXT control treat 3 3 Annotation.TXT”))”.

#### 2.1.8. Determined Shared Up- and Down-Regulated Genes Between DDX5KD and Supinoxin Treatment Data

- In each dataset, up- and down-regulated genes are denoted as follows:
  1. Up-regulated genes—FDR < 0.05 and log2fold-change > 1
  2. Down-regulated genes—FDR < 0.05 and log2fold-change < −1

**Figure 4:**
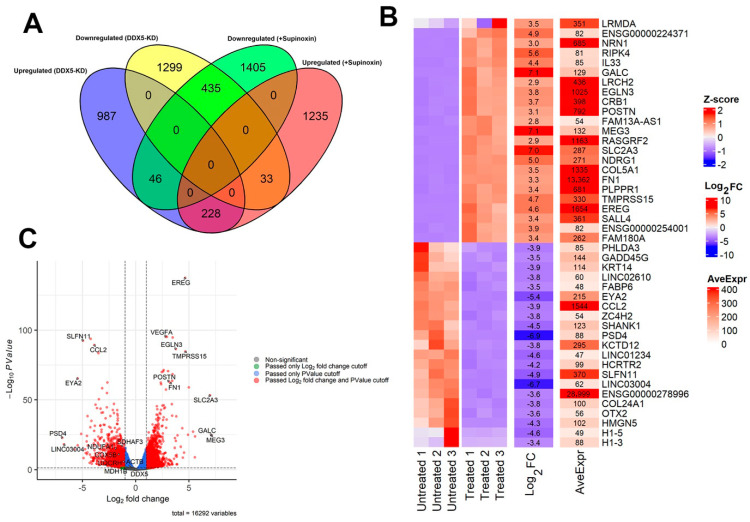
The analysis of RNA-seq data indicates downregulated mitochondrial function following Supinoxin treatment. (**A**) A Venn diagram illustrates the overlap between the total number of up- and down-regulated genes following Supinoxin treatment and DDX5 knockdown. (**B**) Heatmap denoting differential expression patterns for key genes upon Supinoxin treatment. Red and blue colors indicate up- and down-regulation, respectively. From left to right—heatmaps show the expression pattern in untreated samples, Supinoxin-treated samples, log_2_(fold-change) in Supinoxin over untreated and average expression across all samples. (**C**) Volcano plot depicting differentially expressed genes (DEGs) between H69AR cells with or without Supinoxin treatment and key genes are labeled.

A Venn diagram (Figure 4A) was created using the up- and down-regulated genes from each dataset using the ggvenn R-package [38].

#### 2.1.9. Custom Figures

- A heatmap denoting the expression patterns for key genes among Supinoxin-treated and untreated samples was created using the R-package ComplexHeatmap [39] (Figure 4B).
- A heatmap denoting the up- or down-regulation (log_2_ fold change) for key genes in Supinoxin-treated samples as compared to untreated samples was created using the R-package ComplexHeatmap (Figure 4B).
- A heatmap denoting the average expression across Supinoxin-treated and untreated samples was created using the R-package ComplexHeatmap (Figure 4B).
- A volcano plot displaying key differentially expressed genes (−log10 Pvalueon *Y*-axis) along with up- or down-regulation (log_2_ fold change on *X*-axis) in Supinoxin data was created using the R-package EnhancedVolcano (Figure 4C).

#### 2.1.10. Pathway Analysis

Pathway analysis was performed using significant DE genes using the R-package ClusterProfiler.

- Pathway analysis can be performed using various databases such as KEGG, Reactome, Gene Ontology (GO) and others. The choice of database depends on the research question and the specific pathways of interest. For this analysis, we started with Hallmark gene sets from the Molecular Signature Database (MSigDB) via the R-package msigdbr. KEGG OXIDATIVE PHOSPHORYLATION was the top-enriched pathway in the Hallmark gene set.
- To dive deeper into pathway mechanisms and canonical signaling, we did a second-pass analysis with the “C2” curated gene set from MSigDB. Enrichment analysis was performed with pre-ranked genes (Rank = signed fold change × −log10 Pvalue).

Note: The user can specify a different database or gene set collection of choice by updating the relevant function in R-code to perform enrichment analysis with a different database.

- Enrichment analysis for Supinoxin data was performed using the GSEA function available in the clusterProfiler R-package.

Note: The current R-code for the ‘bitr’ function expects the gene IDs as ENSEMBL IDs and converts to ENTREZ IDs as specified by parameters ‘fromType’ and ‘toType’, respectively. The user should update the ‘fromType’ parameter if input gene IDs are of a different type than ENSEMBL IDs. The ‘toType’ parameter should be set as “ENTREZID” as this is the preferred format for subsequent functions in the R-package ClusterProfiler [40].

Note: The ‘GSEA’ function could take a while to run (depending on the processing power of the specific computer). Please be patient.

**Figure 5:**
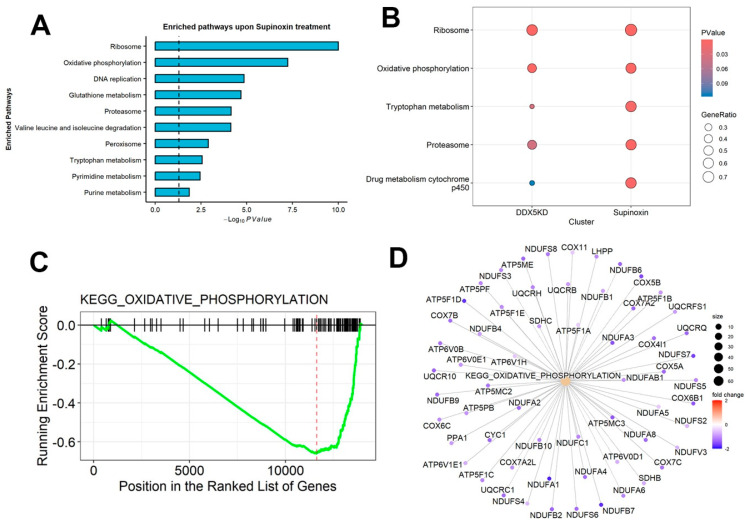
Key pathways affected by Supinoxin treatment. (**A**) Key enriched pathways associated with DEGs in Supinoxin-treated samples are shown. (**B**) Dot plot illustrating comparison of important enriched pathways associated with DDX5 knockdown and Supinoxin treatment. Dot color denotes the significance (*p*-value) and dot size denotes the GeneRatio (number of DEGs detected divided by total genes within the respective pathway). (**C**) Gene set enrichment analysis (GSEA) of the oxidative phosphorylation pathway genes in H69AR +/− Supinoxin-treatment from KEGG collections. (**D**) Gene-concept network plot for the “KEGG Oxidative Phosphorylation” pathway upon Supinoxin treatment. Dot size corresponds to (−log10(*p* Value)).

- A bar plot for important enriched pathways in Supinoxin data was created using GSEA results and the ggplot2 R-package [41] (Figure 5A).
- Enrichment analysis for DDX5 knockdown data was performed using the GSEA function available in the clusterProfiler R-package.
- The compareCluster function from the clusterProfiler R-package was applied to examine biological profiles (reference: C2 database from MSigDB [42]) associated with Supinoxin and DDX5 knockdown genes, and a dot plot displaying simultaneous enrichment of important pathways associated with each treatment was generated (Figure 5B).
- The gseaplot was created to visualize the distribution of the gene set and the enrichment score for KEGG_OXIDATIVE_PHOSPHORYLATION pathway in Supinoxin data (Figure 5C).
- Gene-concept network plot for pathway “KEGG OXIDATIVE PHOSPHORYLATION” in Supinoxin data was created using the cnetplot function from the clusterProfiler R-package (Figure 5D).

#### 2.1.11. Confidence Assessment for the KEGG Oxidative Phosphorylation (OXPHOS) Network

- To assess the robustness of the gene-concept network within the KEGG Oxidative Phosphorylation (OXPHOS) pathway, we determined if transcriptional enrichment (adjusted *p*-value = 3.90 × 10^−6^, normalized enrichment score = −2.11) corresponds to subsequent protein-level organization.
- Gene symbols for the OXPHOS pathway were mapped to corresponding STRING IDs. The majority of genes were successfully mapped and interaction querying yielded greater than 2000 pairwise connections among 60 OXPHOS gene symbols, indicating extensive inter-protein connectivity and a coherent protein interaction network. Visualization of the resulting STRING network was performed to demonstrate a densely interconnected structure with a highly significant *p*-value (Figure S1).
- Each STRING interaction within this network was supported by a combined confidence score metric, which was computed by combining the probabilities from the different evidence channels (e.g., experimental data, conserved neighborhood, Gene fusions, Phylogenetic co-occurrence, Co-expression, Database imports) [43]. STRING combined confidence score ranges from 0 to 1000 (some sources may show a range from 0 to 1, which simply divides the scores by 1000). The combined confidence score was interpreted as (0–400 = low-confidence; 400–700 = high-confidence, and 900–1000 = very high-confidence) [43]. Networks with low-confidence scores generally have more connecting edges, dense networks, and may contain indirect evidence with higher chances of false positives, while networks with high-confidence scores (>700) have fewer edges with strong experimental support and a clean core network with stronger biological evidence. The distribution of STRING confidence scores within the OXPHOS interaction network was examined. As denoted in Figure S1 > 79% interactions within the OXPHOS network have very-high-confidence (>900) scores and the remaining >20% interactions have high-confidence scores (>700), indicating a highly structured and biologically cohesive protein interaction network (Figure S1).
- Next, protein–protein interaction (PPI) enrichment analysis was performed. The OXPHOS network contained 1102 observed edges (ppi_edges) compared to 32 expected edges (ppi_lambda). PPI enrichment analysis *p*-value (ppi_Pvalue) was 0, indicating a highly significant overrepresentation of interactions above random chance. To account for machine precision limits (i.e., *p*-value reported as 0), the smallest positive double value was used to compute a conservative −log10-transformed enrichment metric, yielding a robust quantitative estimate of PPI confidence. Further, a composite network confidence score was computed as a function of OXPHOS pathway enrichment significance, the average of gene-level differential expression strength and PPI enrichment magnitude. High composite network confidence score affirms the significance of the OXPHOS pathway network.
- Next, topological analysis was performed to quantitatively characterize the structural organization of a protein–protein interaction (PPI) network. Topological analysis revealed the organization of protein interactions and associated key genes (termed hub genes) that are strong contributors to network integrity. As denoted in the respective R-code, first a graph object (g) was built from OXPHOS interactions, followed by simplification by removing duplicate edges and self-loops (i.e., removing technically redundant interactions). The resulting graph comprised 60 nodes and 1102 non-redundant edges and a corresponding network density of 0.62 (i.e., 62% of all theoretically possible pairwise interactions were retained within the simplified graph object). Subsequent calculation of the degree of centrality identified nodes with a high number of connections. Nodes with the highest degree of centrality were used to determine the top 10 hub genes that form core components within the OXPHOS network. Visualization of the core OXPHOS network (i.e., protein–protein interactions associated with the top 10 hub genes) was performed.
- Mapping of the OXPHOS network to STRING interactions indicated extensive inter-protein connectivity and >79% interactions with a very high confidence score (>900) denoted biological and functional relevance of protein–protein interactions. Enrichment testing of protein–protein interactions revealed a highly significant *p*-value, confirming that PPI network connectivity exceeds random expectation. Topological analysis moved beyond simple interaction counts and quantitatively characterized the structural organization of a protein–protein interaction network and revealed the hub genes that contribute most strongly to network integrity. Collectively, these analyses indicated that the OXPHOS pathway is not only transcriptionally enriched but also forms a structured, biologically cohesive protein–protein interaction network with hierarchical organization and central hub genes. This reinforced our findings that OXPHOS perturbations are a coordinated pathway-level remodeling activity rather than simple gene-level variations.

### 2.2. Determination of Mitochondrial Respiration by the Seahorse XFe24 Analyzer

#### 2.2.1. Seeding Cells in SEAHORSE XFe24 Plates

Seed H69AR cells in the Seahorse XFe24 Cell Culture Microplate

1. Cell Seeding
  - H69AR is an adherent epithelial small-cell lung carcinoma (SCLC) cell line, derived from a 55-year-old White male patient. It is approximately 50-fold resistant to Adriamycin in comparison to the parental NCI-H69 cell line (ref https://www.atcc.org/products/crl-11351 access on 2 March 2026). The culture conditions of the cell line are RPMI 1640 medium with 20% fetal bovine serum and 1% penicillin-streptomycin solution.
  - Harvest and seed 2 × 10^4^ H69AR cells in 250 µL growth medium (RPMI 1640 medium with 20% fetal bovine serum and 1% penicillin-streptomycin solution) per well. The six background correction wells (A1, A2, D3, D4, D5 and D6) should contain 250 µL of growth media.
  - Gently swirl the plates after adding the cells to ensure uniform distribution of cells across the plate.
  - Rest the plate at 20–25 °C in a tissue culture hood for 1 h to ensure uniform distribution of cells and to minimize edge effects.
  - Incubate the cells for 12–18 h in a cell culture incubator at 37 °C in a humidified environment, supplemented with 5% CO_2_.

Note: Make sure that the background correction wells do not contain any cells.

Note: Optimal cell seeding density differs depending on the cell type, generally ranging from 1 × 10^4^ and 8 × 10^4^ cells per well. Cell densities that achieve 50–90% confluency typically produce metabolic rates within the optimal range of the instrument.

Note: After adding the cells, make sure to swirl them around to ensure a uniform distribution throughout the plate.

Note: While seeding cells, keep in mind to position the pipette tip at the edge of the lower well without touching the bottom of the well to ensure uniform cell distribution.

#### 2.2.2. Addition of Supinoxin and Hydration of Sensory Cartridge

##### Preparation of Supinoxin

- Weigh 22 mg of Supinoxin (ChemieTek, CT-RX5902) powder and dissolve it in 10 mL of 100% DMSO solution to obtain a 5 mM stock solution. Vortex the solution until the powder is fully dissolved.
- Dilute the 5 mM stock solution by mixing equal volumes of the 5 mM Supinoxin solution and DMSO (1:1) to make an intermediate solution of 2.5 mM.
- Prepare a working stock of 175 µM of Supinoxin solution from 2.5 mM solution.
- Add 1 uL of working stock to 2.5 mL growth medium (RPMI 1640 medium with 20% fetal bovine serum and 1% penicillin-streptomycin solution) to obtain a final concentration of 70 nM of Supinoxin.

##### Treatment of H69AR Cells with Supinoxin

- Discard the previous RPMI 1640 medium and add fresh 250 µL of growth medium (RPMI 1640 medium with 20% fetal bovine serum and 1% penicillin-streptomycin solution) supplemented with 70 nM Supinoxin solution to three biological replicates of H69AR cells in triplicate. Similarly, add 250 µL growth medium supplemented with 1 µL DMSO only to three biological replicates of H69AR cells in triplicate as a control.
- Incubate cells for 12–18 h in a cell culture incubator at 37 °C in a humidified environment, supplemented with 5% CO_2_.

Note: The chosen concentration of 70 nM Supinoxin was determined from our previous dose–response experiments conducted in H69AR cells [12]. The cells were treated with varying concentrations of Supinoxin in 96-well plates for 24 h, and cell viability was evaluated using the CyQUANT Direct Cell Proliferation Assay. The IC_50_ value determined from three biological replicates was 69.38 ± 8.89 nM, which is close to 70 nM [1]. When implementing this method in different cell lines or laboratory settings, it is recommended to conduct further dose–response and optimization experiments, since drug sensitivity may differ among cell types and experimental conditions.

##### Hydration of Sensor Cartridge

- Open the Seahorse XFe24 Extracellular Flux Assay Kit, Agilent Technologies, Santa Clara, CA, USA and remove contents inside the cell culture hood.
- Place the sensor cartridge upside down next to the Agilent Seahorse utility plate. Sensor cartridges for Seahorse XF analyzers facilitate sensitive, real-time measurements of cellular metabolic energy pathways. Solid-state sensor probes with polymer-embedded fluorophores create a transient microchamber for the detection of oxygen and proton levels in cell culture medium. The probes are situated around 200 µm above the cells and capture measurements at intervals of a few seconds. The cartridge includes integrated injection ports for the addition of compounds during the assay [44].
- Fill each well of the utility plate with 1 mL of Agilent Seahorse XF Calibrant solution and place the hydro booster (pink, included with the Seahorse XFe24 Extracellular Flux Assay Kit) on the top of the utility plate.
- Lower the sensor cartridge back onto the utility plate gently, through the openings of the hydro booster plates. The hydro booster effectively hydrates solid-state oxygen and pH sensors, resulting in precise outcomes during measurements [45].
- Ensure that the XF calibrant level is sufficiently high to maintain the sensor in a submerged position.
- Incubate the assembled setup in a non-CO_2_ incubator for 12–18 h at 37 °C in a humidified environment.

##### Turning on the Seahorse XFe24 Analyzer

- Turn on the Seahorse XFe24 Analyzer and the computer, then launch the Agilent Seahorse Wave Controller 2.4.1 software.
- Select the ‘‘Heater on” option and allow it to warm overnight.

Note: Check the software to confirm that the temperature is rising towards 37 °C. A green “Connected” icon located in the lower-left corner signifies that the instrument is correctly connected and operating as intended.

#### 2.2.3. On the Day of the Experiment

##### Preparation of Assay Medium

Prepare the assay medium with supplied Seahorse XF RPMI medium, pH 7.4, XF 100 mM pyruvate solution, XF 200 mM glutamine solution, and XF 1 M glucose solution as outlined in Table S1.

##### Washing the H69AR Cells

- Warm the assay medium in a 37 °C water bath before use.
- Take out the XFe24 cell culture microplate from the cell incubator.
- Remove 200 µL of the previous cell growth medium, leaving 50 µL in the well, and replace it with 1 mL of assay medium.
- Repeat the step twice to ensure the complete removal of any traces of the original medium.
- Add 450 µL of assay medium to the cell culture microplate, bringing the final volume to 500 µL.
- Incubate the cell culture microplate in a non-CO_2_ incubator for 1 h at 37 °C to de-gas the plates.

##### Preparation and Injection of XF Cell Stress Compounds

- Take out one foil pouch along with the decapper from the Seahorse XF Cell Mito Stress Test Kit box.
- Remove the test compounds, Oligomycin (blue cap), FCCP (yellow cap) and Rotenone/Antimycin A (Rot/AA) (red cap), from the pouch in a small tube rack.

Note: Exercise extreme caution when handling these compounds, as they are highly toxic.

- Dissolve the dried compounds in the single vial with the assay medium, using the volumes indicated in Table S2.
- Following the addition of the assay media, mix the contents gently by pipetting 10 times to ensure the compounds are solubilized.
- Prepare a final concentration of 1.5 µM oligomycin, 1 µM FCCP, and 0.5 µM Rot/AA by diluting the stock solution of the compounds as indicated in Table S3.
- Remove the hydrated sensor cartridge from the non-CO_2_ incubator.
- Load 56 µL of 1.5 µM oligomycin into injection port A, 62 µL of 1 µM FCCP into injection port B, and 69 µL of 0.5 µM Rotenone/antimycin A into injection port C of the hydrated sensory cartridge.

Note: The optimal final concentration of each compound may differ based on the cell line and the assay medium utilized. It is advisable to conduct a titration experiment for each new cell line or assay medium to identify the optimal concentration.

Note: The injection of the uncoupler, FCCP, induces the maximum respiratory rate. If the oxygen consumption rate (OCR) remains unchanged following FCCP injection, a precise titration experiment is necessary to identify the optimal FCCP concentration.

Note: For Oligomycin, a concentration of 1.5 µM is recommended for most cell types, whereas for Rot/AA, a concentration of 0.5 µM is advised.

##### Running the Assay

- Open the Agilent Seahorse Wave Controller 2.4.1 software in the Seahorse XFe24 analyzer
- Choose the XF Cell Mito Stress Test from the Templates window and configure the program as outlined below.
- To modify the group’s information, click “Add Group” on the plate map section and choose the appropriate wells.
- On the “Protocol” section, the specifications are outlined as follows: Baseline, three cycles; inject port A (oligomycin), three cycles; inject port B (FCCP), three cycles; inject port C (Rotenone/antimycin A), three cycles.
- Each run consists of a 3 min mixing phase, followed by a 2 min wait and concludes with a 3 min measurement.
- On the Run Assay section, click “Start Run” and select a location to save the assay result file.
- The tray will automatically eject and position the calibration plate with the loaded sensor cartridge on the instrument tray. Proceed by clicking “Continue”. Calibration takes about 20 min.
- Prior to positioning the calibration plate with the loaded sensor cartridge on the instrument tray, remove the cartridge lid and confirm the correct orientation of the plate.
- Once the calibration is complete, click “Open the Tray,” substitute the calibration plate with the cell culture microplate, and then click “Start.”

Note: Before starting the assay, ensure that the compounds are injected into the port of the sensory cartridge by carefully inserting the pipette tip to prevent drops on the surface of the cartridge.

##### Data Analysis

- Once the experiment is complete, take out the cell plate and the sensory cartridge.
- Export the results to an external storage device from the system.
- The Seahorse XF Mito Stress Test Report Generator, Agilent Technologies, Santa Clara, CA, USA efficiently computes the parameters of the Seahorse XF Cell Mito Stress Test using Wave data that has been exported as an Excel or Prism File. (https://www.agilent.com/cs/library/usermanuals/public/Report_Generator_User_Guide_Seahorse_XF_Cell_Mito_Stress_Test_Single_File.pdf accessed on 2 March 2026).

## 3. Results

**Figure 6:**
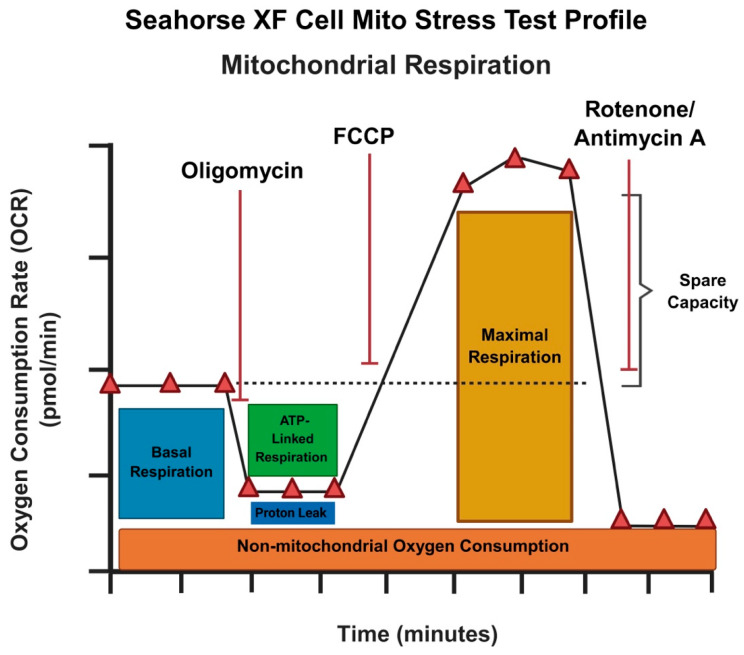
Schematic representation showing Agilent Seahorse XF Cell Mito Stress Test profile. The Seahorse XF Cell Mito Stress Test measures the oxygen consumption rate (OCR) of cells in real time, allowing for the assessment of key mitochondrial function parameters. Created in Biorender.

The analysis of RNA-seq data identified genes that were globally affected upon both DDX5 knockdown and Supinoxin treatment [12]. We compared the list of Supinoxin-responsive genes with our previous RNA-seq data of H69AR cells +/− DDX5 knockdown [11]. The results showed that a total of 435 transcripts (Figure 4A) are significantly downregulated following both Supinoxin treatment or DDX5 knockdown. This overlap suggests that Supinoxin treatment produces effects similar to those observed following DDX5 knockdown in SCLC cells. Subsequently, we identified pathways that were altered to examine the cellular processes affected by Supinoxin. A total of 1299 genes were identified as up-regulated, whereas 1405 genes were identified as down-regulated, corresponding to a total of 152 KEGG pathways. Figure 5A presents the top 10 enriched pathways. DEGs were closely associated with several pathways, including those related to ribosomal components, oxidative phosphorylation, and DNA replication. Importantly, either Supinoxin treatment or DDX5 knockdown results in significant changes in oxidative phosphorylation (OXPHOS) in H69AR cells (Figure 5B). GSEA analysis indicated a significant downregulation of oxidative phosphorylation following Supinoxin treatment, which was accompanied by the downregulation of genes involved in the formation of complexes I-V (Figure 5C). Assessment of the OXPHOS network performed through the STRING database revealed high-confidence protein–protein interactions. Subsequent enrichment analysis showed statistically significant overrepresentation of interactions compared to random chance. Lastly, topological analysis revealed quantitative characteristics of the structural organization of protein–protein interactions by identifying hub (core structural component) genes. These hub genes are components of mitochondrial complex I (*NDUFB6*, *NDUFA5*), complex III (*UQCRQ*, *UQCRH*, *UQCRB*, *UQCRFS1*), complex IV (*COX5A*) and ATP synthesis (*ATP5PF*, *ATP5PB*, *ATP5ME*), indicating coordinated regulation of the electron transport chain and ATP production. Collectively, Supinoxin treatment resulted in significant negative enrichment of the OXPHOS pathway (Figure 5C) with the majority of constituent genes exhibiting reduced expression levels (Figure 5D). Protein–protein interaction analysis revealed high topological connectivity and density within the OXPHOS network (Figure S1), denoting global suppression of mitochondrial respiratory capacity. To test the impact of Supinoxin treatment on mitochondrial function, we conducted a Seahorse XF Cell Mito Stress Test to measure cellular respiration in H69AR cells following Supinoxin treatment [12]. The Seahorse XF Cell Mito Stress Test directly monitors the oxygen consumption rate (OCR) of cells in real time, subsequently evaluating essential aspects of mitochondrial function [46,47,48] (Figure 6). Basal respiration indicates the energy requirements of the cells in normal conditions, where oxygen consumption facilitates ATP production and mitochondrial proton leakage [46]. To evaluate basal oxygen consumption, three measurements of the oxygen consumption rate (OCR) are taken before the injection of any compounds. Oligomycin [49] is first injected into the assay, inhibiting ATP synthase by limiting the flow of electrons through the electron transport chain (ETC), which leads to a decrease in the oxygen consumption rate (OCR). The decrease in OCR correlates with cellular ATP production. The portion of basal respiration that is not associated with ATP production signifies H+ (proton) leak, indicative of mitochondrial damage. The second injection includes FCCP [carbonyl cyanide-p-(trifluoromethoxy) phenylhydrazone] and is utilized to evaluate the maximal respiratory capacity of the cell. FCCP functions as an uncoupler by facilitating proton transport across the mitochondrial membrane, which, in turn, disrupts ATP synthesis [50]. The spare respiratory capacity is defined as the difference between maximal respiration and basal respiration [46]. The spare respiratory capacity indicates the cell’s ability to meet heightened energetic demands. The third injection consisted of a combination of rotenone, a complex I inhibitor, and antimycin A, a complex III inhibitor (Rot/A. The combination inhibits mitochondrial respiration, allowing for the assessment of non-mitochondrial respiration [51]. The analysis indicated a notable reduction in both basal and maximal oxygen consumption rates after Supinoxin treatment. The spare respiratory capacity was also reduced, signifying a decreased ability to fulfill increased energy requirements in SCLC cells [12]. The findings indicate that both Supinoxin treatment and DDX5 knockdown impair mitochondrial activity, leading to decreased cellular respiration in SCLC cells [11,12]. The observed functional alterations align with RNA-seq findings that indicate a suppression of oxidative phosphorylation, which was further confirmed by Seahorse analysis, indicating mitochondrial dysfunction in these conditions.

## 4. Discussion

**Figure 7:**
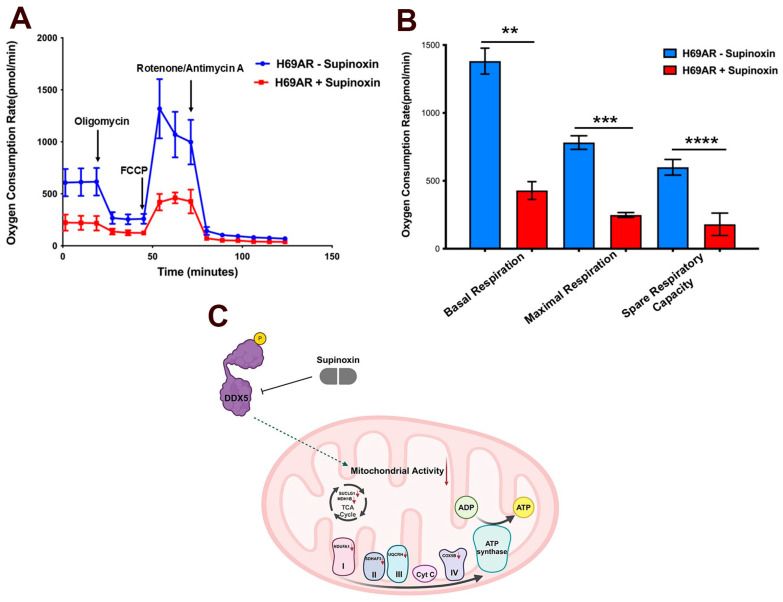
Supinoxin treatment leads to mitochondrial dysfunction in SCLC. (**A**) Line graph and (**B**) bar graph representation of oxygen consumption rates of H69AR cells +/− Supinoxin treatment. Rates were measured using the Seahorse XFe24 metabolic flux analyzer. The data represent the mean ± standard deviation (SD) of three biological replicates. The *p*-values are ** < 0.005, *** < 0.001 and **** < 0.0001; ns, not significant. (**C**) Model depicting the mechanism of Supinoxin in SCLC. (Figure reprinted and adapted with permission from Das et al., 2025 [12]).

In our prior study, we assessed the impact of the small molecule Supinoxin on mitochondrial function in H69AR SCLC cells. To gain insight into the genes affected by Supinoxin treatment on a global scale, RNA-seq was performed on H69AR cells treated with or without Supinoxin. It is crucial to emphasize that both Supinoxin treatment and DDX5 knockdown led to notable changes in oxidative phosphorylation (OXPHOS). GSEA analysis consistently indicated that oxidative phosphorylation is significantly downregulated following treatment with Supinoxin. Next, we examined the rate of mitochondrial respiration by measuring the oxygen consumption rate (OCR) in H69AR cells treated with and without Supinoxin [12]. Results showed a notable reduction in oxygen consumption rates after Supinoxin treatment, suggesting that Supinoxin treatment impacts mitochondrial respiration in H69AR SCLC cells [12] (Figure 7A–C). Prior studies from other laboratories suggested that Supinoxin exerts its effects by interfering with the interaction between phosphorylated DDX5 (pDDX5) and β-catenin [9,22]. However, our results do not fit this model; RNA-seq analysis followed by Seahorse validation indicates that Supinoxin treatment results in mitochondrial dysfunction in H69AR SCLC cells, independent of β-catenin involvement [11,12]. Recent studies indicate that oxidative phosphorylation (OXPHOS) is elevated in various cancer types [52,53,54]. Moreover, OXPHOS inhibition has been shown to reduce the oxygen consumption rate, potentially alleviating tumor hypoxia [55,56,57]. Research findings indicate that lung tumors exhibit elevated oxidative activity and that the development of lung cancer is dependent on oxidative phosphorylation [55,56,57,58]. As there are limited treatment options available for SCLC, this research is significant not just for improving SCLC treatment but also for guiding targeted therapies in various other cancer types.

In general, we expect the bioinformatics steps for RNA-seq data analysis workflow to work seamlessly for any bulk RNA-seq data analysis, provided all input files are correct, commands are accurately tailored for respective sample names with appropriate directory paths and the correct reference genome and annotations. The user should pay careful attention to error messages (if any) and validate that the desired output file/s are successfully generated after each step. In case an output file is not generated, the tool will provide an error message that can be further evaluated. In some scenarios, the output file is generated but could be incomplete (i.e., the output file is generated, but it has incomplete results because the wrong input data was used). Such scenarios of failures are evident in subsequent steps through faulty statistics or errors. For example, if only a single chromosome FASTA file instead of the entire genome is used as a reference, then all the steps will still be completed without any errors, but the output would be limited to only the given chromosome. This type of mistake is evident through subsequent steps, e.g., Mapping statistics will show only a small percentage of reads mapped to the genome OR in the counts matrix will display most genes (except those on the input chromosome) with 0 counts. Therefore, it is critical to carefully inspect the output files generated after each step. Please refer to Table 2 for acceptance thresholds for key parameters and statistics within the RNA-seq workflow, along with suggestions for corrective actions. Replicates are essential for any RNA-seq analysis, and at least three replicates per group are recommended by ENCODE. Our article includes guidelines to assess the replicate concordance and guidelines to handle sparse (low correlation) replicates. This includes example code for different visualizations (Venn diagram, heatmap, volcano plot) using DE results and can be tailored for other RNA-seq data with minor modifications. Post-differential expression analysis, pathways can be determined using multiple databases, and the choice of databases is dependent on the research questions or hypothesis, as well as personal preference. Example code can be easily tailored to different pathway databases with minor modifications. After the pathway of interest is identified, subsequent analysis using the STRING database can be applied to determine the robustness of the network through assessment of protein–protein interactions. In summary, the RNA-seq analysis below provides a highly customizable end-to-end workflow and can be easily adapted to analyze any bulk RNA-seq datasets.

**Figure 8:**
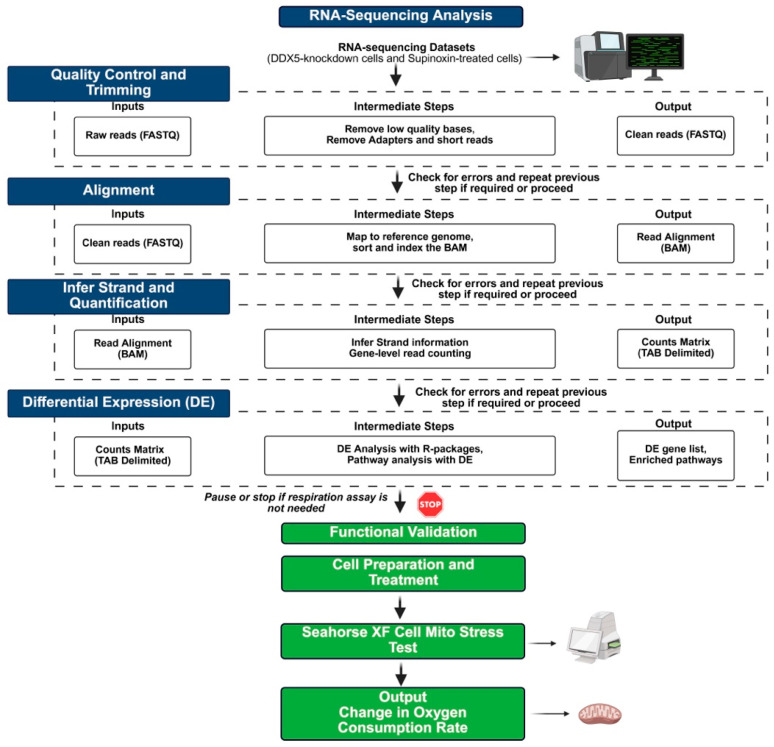
A comprehensive experimental workflow that depicts the inputs, intermediate analytical steps, and outputs of the integrated RNA-seq and Seahorse functional analysis pipeline. RNA-seq data are subjected to quality control, alignment, quantification, and differential expression analysis to produce gene and pathway outputs. Following transcriptomic analysis, users may either end their investigation or advance to functional validation, utilizing Seahorse XF assays to assess alterations in cellular oxygen consumption, thereby independently assessing metabolic effects.

Finally, RNA-seq analysis combined with Seahorse-based validation suggests that Supinoxin leads to mitochondrial dysfunction in H69AR SCLC cells. This article distinctly emphasizes two experimental approaches—RNA-seq and Seahorse-based metabolic analysis that converge to a common mechanistic insight (Figure 8). This research is significant not just for improving SCLC treatment but also for guiding targeted therapies in various other cancer types.

## 5. Troubleshooting

Problem 1

If the percentage of mapped reads to the reference genome is less than 80%.

Potential Solution

A low percentage of mapped reads could be caused by low data quality (low sequencing quality, adapters not trimmed, short reads) or contamination. Make sure to check the quality statistics before and after trimming. Quality statistics from FastQC (e.g., total number of reads retained after quality trimming, any adapters remaining after trimming, and the overall sequence quality and read length distribution) are very informative for identifying issues. Trimming parameters can then be adjusted accordingly. If the quality is appropriate, a contamination screening can be performed through tools like DeconSeq or FastQ Screen, followed by the removal of unwanted sequences.

Problem 2

Low percentage of reads mapping to genes/transcripts.

Potential Solution

Make sure to determine the strandedness of RNA-seq data (step 3) and set appropriate strand information while qualification (step 4). For example, if a strand-specific parameter is set for un-stranded data, then only 50% of reads (from forward or reverse strand) will be counted in the genes. Therefore, inferring appropriate data strandedness is a crucial step.

Problem 3

Re-analysis of RNA-seq data with different software versions.

Potential Solution

As new versions of software packages are released, any update in the underlying methods or databases can lead to variations in the results. A publication may have included exact software versions, but it may not be feasible to get these installed on a system. This is a common and not surprising situation. Re-analysis using different cut-offs, different program versions, parameters, etc., can give different numbers, but overall findings and biological conclusions should be the same.

Problem 4

Significant variability among replicates in Seahorse XF Cell Mito Stress Test.

Potential Solution

The high variation between replicates can be attributed to uneven cell seeding or the sensor’s inability to accurately detect signals.

Cell Seeding: Ensure that the appropriate volume of cells is added to each well of the cell plate. Inconsistent results may also arise from uneven cell distribution. Prior to the start of the experiment, it is essential to test and optimize the seeding density for each cell type.

Hydration of the Sensory Cartridge: Ensure that the sensors are hydrated for 12–18 h with an adequate volume of XF Calibrant in the non-CO_2_, 37 °C incubator, and utilize sensor cartridges and cell microplates prior to their expiration date. If the sensory cartridge is not adequately hydrated, it may result in experimental failure, causing incorrect OCR values.

Problem 5

Poor basal signal during Seahorse XF Cell Mito Stress Test.

Potential Solution

The poor basal signal may be attributed to the inadequate number of cells. To prevent this, increase the number of cells per well. Another factor responsible for inadequate basal signal could be the prolonged incubation of the cell microplate in a non-CO_2_, 37 °C incubator. This will reduce the viability of cells. Ensure that the incubation time for the cell plate is in a non-CO_2_, 37 °C incubator for a maximum of 1 h and no longer.

Problem 6

Minimal or unexpected changes in OCR following drug injection.

Potential Solution

Improper drug injection: Ensure that all drugs are injected directly into the appropriate ports in the correct orientation, avoiding contamination of other ports.

The concentration of the drug injected is too low for a specific cell type. To address this issue, it may be necessary to perform a titration of the drug concentration.

Inconsistent drug injections across wells: Ensure that all designated injection ports are correctly loaded with the appropriate drugs in specified volumes to maintain consistent, balanced injections.

Improper alignment of the cartridge or cell plate while plating or conducting the assay: If the cartridge is oriented incorrectly, the drugs will be injected in the wrong order. Make sure the cell plate and the cartridge are oriented correctly during the experiment.

Problem 7

The XFe24 Analyzer does not include a cooling function; the instrument tends to reach temperatures of approximately 25°C to 26 °C during operation.

Potential Solution

This issue can be addressed by placing the Seahorse Analyzer in a controlled environment for temperature and humidity, or by ensuring that the lab temperature remains at 20 °C [47].

## 6. Statistical Analysis

We recommend following ENCODE standards as minimum requirements in terms of sample size, average library insert size, sequencing-depth, number of replicates, and replicate concordance. Typical recommendations for bulk RNA-seq include: the average library insert size of 200 bp, three or more replicates per group, 30 million aligned reads for each replicate, and a Spearman correlation of >0.9 between isogenic replicates and >0.8 between anisogenic replicates (i.e., replicates from different donors). Our RNA-seq workflow on GitHub (https://github.com/sagarutturkar/RNAseq.Seahorse.Validation.TranLab2025 accessed on 2 March 2026) provides steps to calculate the correlation between replicates. In general, three replicates per group is a widely accepted standard for bulk RNA-seq experiments and provides adequate power to detect biologically meaningful fold-changes while balancing cost and feasibility [59,60]. A systematic study testing 48 biological replicates determined that three replicates per group have reasonable power to reliably detect large fold-changes (>4×), six replicates per group have power to reliably identify >2× fold-changes, whereas an even greater number of replicates are needed for high variability (e.g., heterogeneous clinical samples) [60].

Figure 7 was plotted using Prism 9.0 (version 9.3.0). Data is presented as mean ± SD. Comparisons between two groups were performed using Student’s *t*-test. *p*-values are indicated as ** < 0.005, *** < 0.001 and **** < 0.0001; ns, not significant. This study was conducted with three independent biological replicates (n = 3).

## 7. Conclusions

Here, we provide methods for the integration of two separate methodologies: RNA-seq and Seahorse-based functional validation. Methods that have been published before concentrate solely on either RNA-Seq or Seahorse analysis. This article is distinct as it outlines a detailed procedure to elucidate the mechanism of action of Supinoxin in relation to SCLC. Initially, we utilized RNA-seq to thoroughly examine alterations in gene expression, uncovering that oxidative phosphorylation is impacted in both DDX5KD and Supinoxin-treated H69AR cells. To validate these findings functionally, we conducted Seahorse-based metabolic analysis, which directly assessed mitochondrial respiration after drug treatment. SCLC cells demonstrate a high metabolic demand, and DDX5 activity is essential for these cells to sustain mitochondrial respiration [11]. The depletion of DDX5 notably disrupts mitochondrial function, consequently impacting cellular respiration [11]. Studying metabolic activity is essential in SCLC cells following DDX5 knockdown and Supinoxin treatment [11,12]. This comprehensive approach demonstrated that Supinoxin disrupts mitochondrial respiration in SCLC cells, corroborating the RNA-Seq findings and uncovering a new mechanism of action. In conclusion, the combination of RNA-Seq and Seahorse functional validation provides a deeper insight into the DDX5-dependent metabolic activity and subsequent therapeutic approaches targeting metabolic activity in SCLC.

## Figures and Tables

**Table 1:** Chemicals, Software and Deposited Data Table.

Reagent or Resource	Source	Reference
**Chemicals**		
RPMI 1640 medium	ATCC, Manassas, VA, USA	30-2001
Penicillin-Streptomycin (10,000 U/mL)	Gibco, Life Technologies Grand Island, NY, USA	15140122
Fetal Bovine Serum—Premium	Biotechne (R&D systems), Minneapolis, MN, USA	S11150
Supinoxin (RX-5902)	ChemieTek, Indianapolis, IN, USA	CT-RX5902
TrypLE™ Express Enzyme (1X), phenol red	Gibco, Life Technologies, Grand Island, NY, USA	12605010
Seahorse XF RPMI Medium pH 7.4	Agilent Technologies, Santa Clara, CA, USA	103576-100
Seahorse XF Calibrant pH 7.4	Agilent Technologies, Santa Clara, CA, USA	100840-000
Seahorse XF 200 mM Glutamine Solution	Agilent Technologies, Santa Clara, CA, USA	103579-100
Seahorse XF 100 mM Pyruvate Solution	Agilent Technologies, Santa Clara, CA, USA	103578-100
Seahorse XF 1.0 M Glucose Solution	Agilent Technologies, Santa Clara, CA, USA	103577-100
**Critical commercial assays**		
Seahorse XF Cell Mito Stress Test Kit	Agilent Technologies, Santa Clara, CA, USA	103015-100
Seahorse FluxPaks	Agilent Technologies, Santa Clara, CA, USA	102342-100
PCR Mycoplasma Detection Kit	Applied Biological Mat. Inc., Richmond, BC, Canada	G238
**Deposited data**		
RNA Sequencing	NCBI Gene Expression Omnibus (Das et al. [12])	GSE255741
RNA Sequencing	NCBI Gene Expression Omnibus (Xing et al. [11])	GSE142024
**Experimental models: Cell lines**		
NCI-H69AR (H69AR)	ATCC, Manassas, VA, USA	CRL-11351
**Software and algorithms**		
GraphPad Prism	GraphPad	version 9.3.0
Seahorse Wave	Agilent Technologies, Santa Clara, CA, USA	https://www.agilent.com/cs/library/usermanuals/public/Report_Generator_User_Guide_Seahorse_XF_Cell_Mito_Stress_Test_Single_File.pdf (accessed on 2 March 2026)
Biorender	Biorender	https://www.biorender.com
fastqc	https://www.bioinformatics.babraham.ac.uk/projects/fastqc/ (accessed on 2 March 2026)	version 0.23.2
fastp	https://github.com/OpenGene/fastp (accessed on 2 March 2026)	version 0.12.1
STAR aligner	https://github.com/alexdobin/STAR (accessed on 2 March 2026)	version 2.7.11b
samtools	https://www.htslib.org/download/ (accessed on 2 March 2026)	version 1.17
rseqc	https://rseqc.sourceforge.net/	version 4.0.0
Subread (featurecounts)	https://subread.sourceforge.net/	version 2.0.1
csvtk	https://github.com/shenwei356/csvtk (accessed on 2 March 2026)	version 0.25.0
tpmcalculator	https://github.com/ncbi/TPMCalculator (accessed on 2 March 2026)	version 0.0.4
R-software	the R Core Team and the R Foundation for Statistical Computing	version 4.3.1
R-package DESeq2	https://www.bioconductor.org/packages/release/bioc/html/DESeq2.html (accessed on 2 March 2026)	version 1.42.1
R-package ggplot2	https://ggplot2.tidyverse.org/	Version 3.5.1
R-package gplots	https://cran.r-project.org/web/packages/gplots/index.html (accessed on 2 March 2026)	version 3.2.0
R-package tidyverse	https://www.tidyverse.org/	version 2.0.0
R-package RColorBrewer	https://cran.r-project.org/web/packages/RColorBrewer/index.html (accessed on 2 March 2026)	version 1.1-3
R-package edgeR	https://www.bioconductor.org/packages/release/bioc/html/edgeR.html (accessed on 2 March 2026)	version 4.0.16
R-package ggrepel	https://ggrepel.slowkow.com/	version 0.9.6
R-package ComplexHeatmap	https://www.bioconductor.org/packages/release/bioc/html/ComplexHeatmap.html (accessed on 2 March 2026)	version 2.18.0
R-package dplyr	https://dplyr.tidyverse.org/	version 1.1.4
R-package EnhancedVolcano	https://bioconductor.org/packages/release/bioc/html/EnhancedVolcano.html (accessed on 2 March 2026)	version 1.20.0
R-package circlize	https://cran.r-project.org/web/packages/circlize/index.html (accessed on 2 March 2026)	version 0.4.16
R-package msigdbr	https://igordot.github.io/msigdbr/ (accessed on 2 March 2026)	version 7.5.1
R-package clusterProfiler	https://bioconductor.org/packages/release/bioc/html/clusterProfiler.html (accessed on 2 March 2026)	version 4.10.1
R-package org.Hs.eg.db	https://bioconductor.org/packages/release/data/annotation/html/org.Hs.eg.db.html (accessed on 2 March 2026)	version 3.18.0
R-package ggvenn	https://cran.r-project.org/web/packages/ggvenn/readme/README.html (accessed on 2 March 2026)	version 0.1.10
R-package openxlsx	https://cran.r-project.org/web/packages/openxlsx/index.html (accessed on 2 March 2026)	version 4.2.7.1
**Others**		
Multichannel pipettors for 20–1000 μL	n/a	n/a
Seahorse XFe24 extracellular flux analyzer	Seahorse Biosciences, Agilent Technologies, Santa Clara, CA, USA	n/a
Cell Counter	n/a	n/a

**Table 2:** Acceptance thresholds for key quality metrics and mapping parameters, along with recommendations for corrective actions when these criteria are not met.

Parameter	Recommended Threshold	Rationale and Corrective Actions
Median Phred Per-base quality	Q30	Phred score Q30 denotes 0.1% error probability. Including bases below Q30 is not catastrophic but it includes noisy data into analysis and may impact alignment scores.
Minimum read length after trimming	50 bp	Dropping the cutoff includes shorter reads into alignment steps and may have a negative impact in terms of ambiguous mapping, inflated multi-mapping reads, and false positives in gene expression.
Adapter content after trimming	0–5%	Lower adapter content is better, but small leftover fractions are common. Higher adapter contents have negative implications as the adapter does not match the genome, causing mismatches at the 3′ end and lowering the overall mapping quality.
Duplication rates	10–30%	RNA-seq naturally has higher duplication due to highly expressed genes. Duplicates should NOT be removed. However, >30% duplication is concerning, and there is a trade-off of accepting the data with caveats or may need to redesign the library preparation.
Mapping rate	80–90%	Typically, >80% mapping rate is acceptable, >85% is good and >90% is excellent. For low mapping rates, primary checks should be verifying the appropriate genome and annotations, verifying data quality before/after trimming, and finally checking for library contamination using tools like FastQ-screen [29].

## Data Availability

•RNA-seq data (GEO Database: GSE255741 and GSE142024) from the NCBI Gene Expression Omnibus database were used for analysis. The accession number is listed in the Key Resources table. The extended version of the code used in this article is provided on GitHub (https://github.com/sagarutturkar/RNAseq.Seahorse.Validation.TranLab2025 (accessed on 2 March 2026)). A stable version of the differential expression analysis script is available on Zenodo with DOI: https://doi.org/10.5281/zenodo.18638583. Any additional information required to reanalyze the data reported in this paper is available from the lead contact upon request.

## Supplementary Materials

The following supporting information can be downloaded at: https://www.mdpi.com/article/10.3390/mps9020046/s1, Statistical Table S1: Statistical analysis data related to Figure 7A,B; Statistical Table S2: Statistical analysis data, related to Figure 4 and Figure 5; Parameters Table S3: Key parameters table related to RNA-seq analysis; Table S1: Preparation of Assay Medium; Table S2: Preparation of stock solutions of oligomycin, FCCP and Rot/AA; Table S3: Preparation of working concentrations of oligomycin, FCCP and Rot/AA; Figure S1: Confidence Assessment of the OXPHOS network as a targeted pathway of Supinoxin: A. Visualization of protein-protein interactions (within the STRING database) associated with differentially expressed genes within OXPHOS network upon Supinoxin treatment (Pvalue = 0) B. Distribution of confidence scores assigned to OXPHOS protein-protein interactions show that >79% of the interactions have a very high (>900) confidence score in the STRING database. C. Visualization of protein-protein interactions for the top 10 hub genes, which form a core within the OXPHOS network (*p* value = 0); Data S1: Gene expression counts of DDX5 knockdown from RNA-seq analysis, related to Figure 4 and Figure 5; Data S2: Gene expression counts of Supinoxin treatment from RNA-seq analysis, related to Figure 4 and Figure 5; Supplement S1.pdf—complete R-code for RNAseq analysis.
