# Supplementary material for: Investigating Metabolically Altered Pathways in Small Cell Lung Cancer: From RNA Sequencing Analysis to Seahorse-Based Functional Validation
Source: Methods Protoc. 2026 Mar 10;9(2):46. doi: 10.3390/mps9020046 (PMC13010649; doi:10.3390/mps9020046)
Supplement: Supplementary file 1 [file mps-09-00046-s001.zip › Supplementary Information_EJT/Figure S1 Table S1-S3.pdf]

Table S1: Preparation of Assay Medium

Reagent	Final Concentration	Amount
100 mM pyruvate solution	1 mM	1 mL
200 Mm glutamine solution	2 mM	1 mL
1 M glucose solution	10 mM	1 mL
Seahorse XF RPMI medium	-	97 mL

Table S2: Preparation of stock solutions of oligomycin, FCCP and Rot/AA

Compound	Amount of assay medium	Stock solution
Oligomycin	630 µl	100 µM
FCCP	720 µl	100 µM
Rot/AA	540 µl	50 µM

Table S3: Preparation of working concentrations of oligomycin, FCCP and Rot/AA

Compound	Stock solution to be added	Amount of assay medium	Final Concentration
Oligomycin	450 µl	2.55 ml	1.5 µM
FCCP	300 µl	2.70 ml	1 µM
Rot/AA	300 µl	2.70 ml	0.5 µM

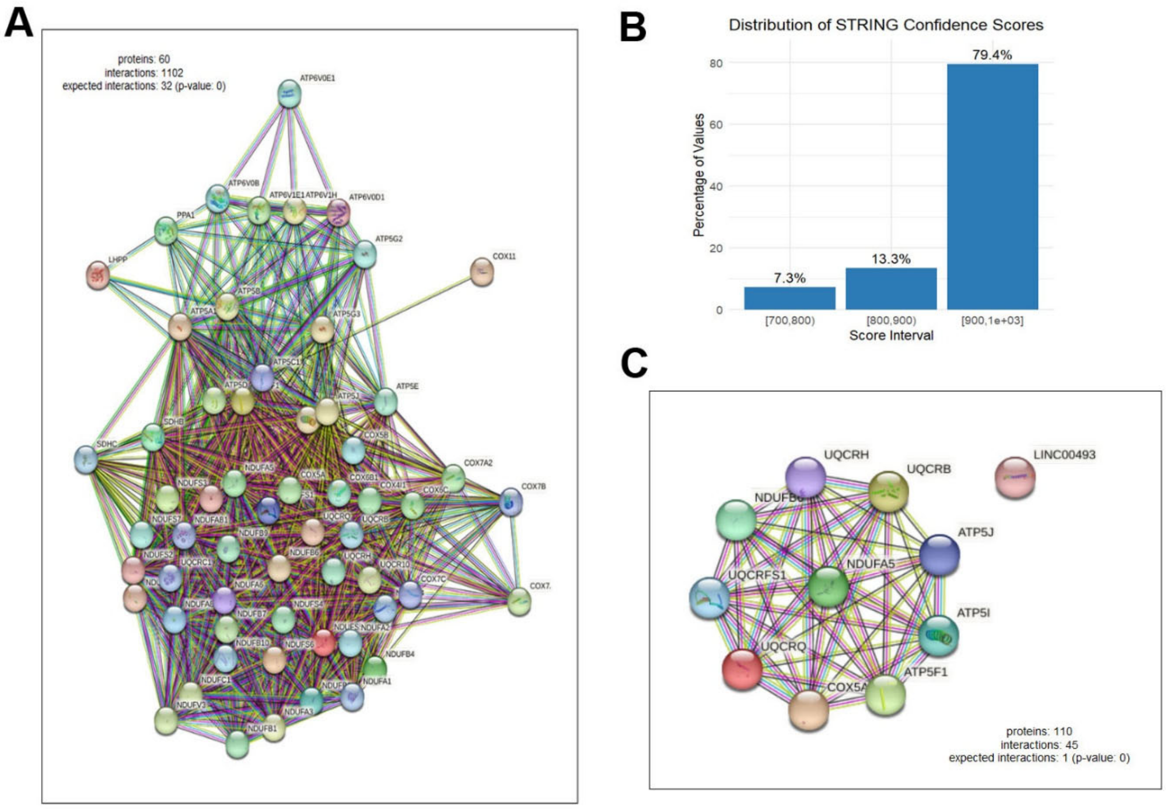

Figure S1: Confidence Assessment of the OXPHOS network as a targeted pathway of Supinoxin: **A.** Visualization of protein-protein interactions (within the STRING database) associated with differentially expressed genes within OXPHOS network upon Supinoxin treatment (Pvalue = 0) **B.** Distribution of confidence scores assigned to OXPHOS protein-protein interactions show that >79% of the interactions have a very high (>900) confidence score in the STRING database. **C.** Visualization of protein-protein interactions for the top 10 hub genes, which form a core within the OXPHOS network (Pvalue = 0).
