# Supplementary material for: Investigating Metabolically Altered Pathways in Small Cell Lung Cancer: From RNA Sequencing Analysis to Seahorse-Based Functional Validation
Source: Methods Protoc. 2026 Mar 10;9(2):46. doi: 10.3390/mps9020046 (PMC13010649; doi:10.3390/mps9020046)
Supplement: Supplementary file 1 [file mps-09-00046-s001.zip › Supplementary Information_EJT/Figure S1.pdf]

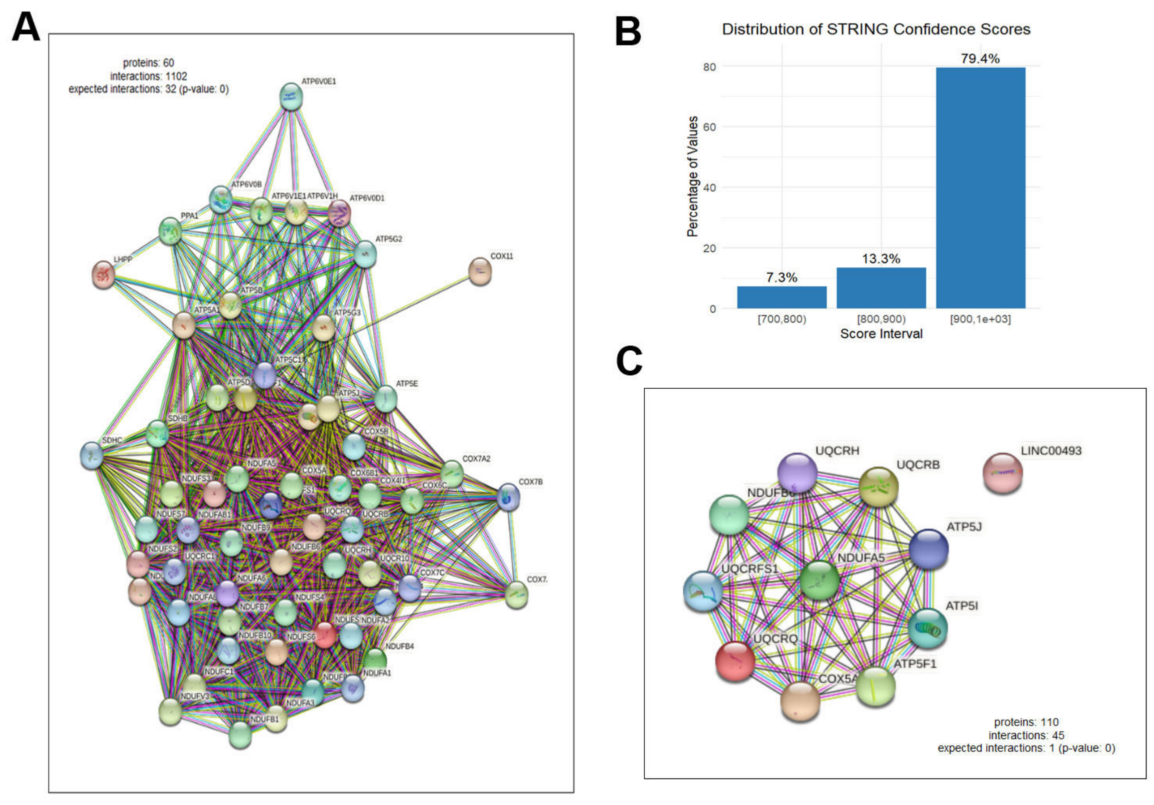
A
proteins: 60
interactions: 1102
expected interactions: 32 (p-value: 0)
B
Distribution of STRING Confidence Scores
Percentage of Values
Score Interval
7.3%
13.3%
79.4%
[700,800)
[800,900)
[900,1e+03]
C
proteins: 110
interactions: 45
expected interactions: 1 (p-value: 0)
UQCRH
UQCRB
LINC00493
ATP5J
NDUFA5
UQCRFS1
ATP5I
UQCRQ
ATP5F1
