# Supplementary material for: Investigating Metabolically Altered Pathways in Small Cell Lung Cancer: From RNA Sequencing Analysis to Seahorse-Based Functional Validation
Source: Methods Protoc. 2026 Mar 10;9(2):46. doi: 10.3390/mps9020046 (PMC13010649; doi:10.3390/mps9020046)
Supplement: Supplementary file 1 [file mps-09-00046-s001.zip › Supplementary Information_EJT/Supplement 1.pdf]

Methods and Protocols - TranLab (Purdue University) 2026

Sagar Utturkar

2026-02-14

- Pre-requisites:
  - Hardware:
  - Linux based software/tools:
  - R-packages:
- Step 1: Preparation: day 1
  - Create working directory:
  - Download reference data:
- Step 2: Quality control of FASTQ data: day 1
  - a. Quality assessment
  - b. Quality-based trimming
- Step 3: Indexing reference genome: day 1
  - a. Indexing:
- Step 4: Alignment with reference genome: day 2
  - a. Mapping:
- Step 5: Infer data strandedness: day 3
  - running infer_experiment.py script:
- Step 6: Quantification to generate counts matrix: day 3
  - Counts matrix in RNAseq:
  - combine counts:
  - Transcripts Per Million (TPM) counts:
  - TPM counts matrix generation:
  - Determine correlation between replicates:
    - Recommendations for low correlation among replicates:
- Step 7: Differential Expression (DE) analysis: day 4
  - DE_edger.R script for differential gene expression:

- Step 8: Determine shared up- and down-regulated genes between DDX5KD and Supinoxin data: day 4
  - Up- and down-regulated genes:
  - Venn diagram **(Figure 1A)** for up- and down-regulated genes:
- Step 9: Custom figures: day 4
  - Heatmap **(Figure 1B)** with individual replicates:
  - Heatmap **(Figure 1B)** with log2FoldChnage data:
  - Heatmap **(Figure 1B)** with Average Expression data:
  - Volcanoplot **(Figure 1C)**:
- Step 10: Pathway analysis: day 4
  - Database for pathway analysis:
  - Enrichment analysis for Supinoxin data:
  - Supinoxin barplot **(Figure 2A)**:
  - Enrichment analysis for DDX5 knockdown data:
  - compareCluster **(Figure 2B)**:
  - Supinoxin gseaplot **(Figure 2C)**:
  - Supinoxin cnetplot **(Figure 2D)**:
- Step 11: Confidence Assessment for KEGG Oxidative Phosphorylation (OXPHOS) Network:
  - Load STRING Database:
  - Mapping OXPHOS genes to STRING Database:
  - Assessment of OXPHOS protein-protein interactions:
  - Enrichment analysis of OXPHOS protein-protein interactions:
  - Topological analysis of OXPHOS protein-protein interactions:
  - Conclusion:

Pre-requisites:

Hardware:

```
This pipeline is primarily designed for execution on Linux based c
Most of the protocol steps can be executed on a modern personal co
equipped with an Intel Core i7 processor and 16 GB of memory. Howe
alignment step, it is highly recommended to utilize a High-Perform
```

```
cluster. Using the HPC cluster likely reduce the processing time f
days to hours based on specific tasks.

We have enlisted minimum and recommended hardware requirements for

Expected data:
Bulk RNA-seq (typical 30–50 Million reads/sample, ~10 samples)

Miminum hardware:
  1. CPU: 8 cores
  2. RAM: 32–64 GB
  3. Disk: ≥ 200 GB (HDD for storage, SSD for fast I/O is recommen
  4. OS: Linux (Ubuntu, CentOS, etc.)

Recommended hardware:
  1. CPU: 16-32 cores
  2. RAM: 128 GB
  3. Disk: ≥ 500 GB (HDD for storage, SSD for fast I/O is recommen
  4. OS: Linux (Ubuntu, CentOS, etc.)
```

Linux based software/tools:

```
# We expect following tools available/installed with Linux-based s
# We refer to each tool as module and loaded below with command 'm

module load fastp
module load fastqc
module load star
module load samtools
module load subread
module load rseqc
module load csvtk
module load tpmcalculator
```

R-packages:

```
library(DESeq2)
library(ggplot2)
library(gplots)
library(tidyverse)
library(RColorBrewer)
```

```
library(edgeR)
library(ggrepel)
library(ComplexHeatmap)
library(dplyr)
library(EnhancedVolcano)
library(circlize)
library(msigdbr)
library(clusterProfiler)
library(org.Hs.eg.db)
library(ggvenn)
library(openxlsx)
library(corrplot)
```

Step 1: Preparation: day 1

Timing: 2 hours

Create working directory:

```
# All analysis should be carried out in a specific directory.
# Here we define a base directory as "RNAseq_Analysis" and
# all paths are in reference to this base directory.

cd ~
mkdir RNAseq_Analysis
```

Download reference data:

Raw FASTQ files were obtained from GSE255741 (Supinoxin) and GSE142024 (DDX5). Reference human genome sequence (hg38) in FASTA format and annotation in GTF format was downloaded from Ensembl genome browser (https://www.ensembl.org/). BED format gene annotations for Human (hg38_GENCODE_V47.bed.gz) were downloaded from (https://sourceforge.net/projects/rseqc/files/BED/Human_Homo_sapiens/).

```
cd ~/RNAseq_Analysis/

mkdir input

# Copy or download the paired-end FASTQ data.
```

```
# For this instruction, we assume data is labelled as:
# control1_1.fastq.gz  and  control1_2.fastq.gz

##########

mkdir reference
cd reference

wget https://ftp.ensembl.org/pub/release-114/fasta/homo_sapiens\
/dna/Homo_sapiens.GRCh38.dna.toplevel.fa.gz

gunzip Homo_sapiens.GRCh38.dna.toplevel.fa.gz
mv Homo_sapiens.GRCh38.dna.toplevel.fa genome_ref.fasta

##########

cd ~/RNAseq_Analysis/

mkdir annotations
cd annotations

wget https://ftp.ensembl.org/pub/release-114/gtf/\
homo_sapiens/Homo_sapiens.GRCh38.114.chr.gtf.gz

gunzip Homo_sapiens.GRCh38.114.chr.gtf.gz
mv Homo_sapiens.GRCh38.114.chr.gtf genome_ref.gtf

##########

wget https://sourceforge.net/projects/rseqc/files/BED/\
Human_Homo_sapiens/hg38_GENCODE_V47.bed.gz

gunzip hg38_GENCODE_V47.bed.gz
sed -i 's/^chr//g'  hg38_GENCODE_V47.bed
```

> **Note:** We have included a specific version (release-114) of human genome in our download link above to ensure reproducibility. The genome assembly and annotations are frequently updated, and it is recommended to use the latest version of assembly and annotations. The underlaying biology and pathways should not change but

> results for individual genes, counts and significance may have changes pertaining to annotation updates.

> **Note:** The RNAseq data used in current analysis were published earlier. While earlier publications included key RNAseq results (differential-expression, top enriched pathways) and brief methods, end-to-end workflow to analyze RNAseq data was not included. The current publication includes step-by-step workflow with detailed instructions and working code to reproduce the results. This end-to-end RNAseq workflow will serve as a primer to perform standard RNAseq analysis on other relevant datasets.

Step 2: Quality control of FASTQ data: day 1

Timing: up to 4 hours per sample

a. Quality assessment

Quality assessment for each data was performed using fastqc tool.

```
cd ~/RNAseq_Analysis/

mkdir quality_control
cd quality_control

mkdir fastqc_before
cd fastqc_before

mkdir control1

fastqc ~/RNAseq_Analysis/input/control1_1.fastq.gz -t 30  -o contr
fastqc ~/RNAseq_Analysis/input/control1_2.fastq.gz -t 30  -o contr

# Repeat above steps for every FASTQ file for each sample
```

b. Quality-based trimming

Quality-based trimming (removal of adapters, low quality bases and short sequences) fastp. After trimming, re-assessment of trimmed data was performed using fastqc to ensure optimal data quality.

```
cd ~/RNAseq_Analysis/

mkdir fastp
cd fastp

# Processing for sample control1

fastp -w 30 \
-i ~/RNAseq_Analysis/input/control1_1.fastq.gz \
-I ~/RNAseq_Analysis/input/control1_2.fastq.gz \
-o control1_1.trimmed.fastq.gz \
-O control1_2.trimmed.fastq.gz \
--length_required  50 \
-q 30 \
--detect_adapter_for_pe  \
-h control1.html  \
-j control1.fastp.json


cd ~/RNAseq_Analysis/quality_control/

mkdir fastqc_after
cd fastqc_after

mkdir control1.trimmed

fastqc ~/RNAseq_Analysis/quality_control/fastp/control1_1.trimmed.
-t 30 \
-o control1.trimmed

fastqc ~/RNAseq_Analysis/quality_control/fastp/control1_2.trimmed.
-t 30 \
-o control1.trimmed

# Repeat above steps for each sample
```

Step 3: Indexing reference genome: day 1

Timing: 4 hours

a. Indexing:

Indexing of reference genome (structured representation of a genome to enable faster and more efficient searching and alignment of DNA sequences) was performed through STAR aligner.

```
cd ~/RNAseq_Analysis/

cd reference

STAR --runThreadN 30 \
--runMode genomeGenerate \
--genomeDir . \
--genomeFastaFiles genome_ref.fasta
```

Step 4: Alignment with reference genome: day 2

Timing: up to 6 hours per sample

a. Mapping:

a. Quality trimmed reads were mapped to reference genome using the STAR aligner. Alignment summary file was inspected to ensure a suitable percentage of reads are mapped to reference genome. The aligned data (BAM format) was generated for each sample.

```
cd ~/RNAseq_Analysis/

mkdir mapping
cd mapping
```

```
STAR --runThreadN 30 \
--runMode alignReads \
 --outSAMunmapped Within \
--outSAMattrIHstart 0 \
--outFilterIntronMotifs RemoveNoncanonical  \
--genomeDir ~/RNAseq_Analysis/reference/ \
--readFilesIn ~/RNAseq_Analysis/quality_control/fastp/control1_1.t
~/RNAseq_Analysis/quality_control/fastp/control1_2.trimmed.fastq.g
--readFilesCommand zcat \
--twopassMode Basic \
--outSAMtype BAM SortedByCoordinate \
--outFileNamePrefix control1.

# --outFilterIntronMotifs RemoveNoncanonical prioritizes robust ge

# --twopassMode Basic increases splice junction accuracy and sensi

samtools sort -@ 30 -n  control1.Aligned.sortedByCoord.out.bam  -c
samtools index -@ 30   control1.Aligned.sortedByCoord.out.bam
samtools flagstat -@ 30 control1.Aligned.sortedByCoord.out.bam > c

# Repeat above steps for each sample
```

> **Note:**
>
> `--outFilterIntronMotifs RemoveNoncanonical` option in STAR aligner is used to remove non-canonical splice junctions from the output. Non-canonical splice junctions were filtered out to reduce alignment artifacts and improve the robustness of gene-level quantification. Rare canonical introns (GC–AG, AT–AC) were retained. While noncanonical splicing events may be biologically relevant in cancer, they are often associated with splicing errors and can introduce noise in the data. Noncanonical splicing events are typically rare and require junction-level validation. This was beyond the scope of this study and filtering was appropriate for our (gene-level) analysis goals.

> `--twopassMode Basic` option in STAR aligner is used to perform a two-pass alignment. In the first pass, STAR identifies splice junctions from the initial alignment. In the second pass, STAR re-aligns the reads to augmented junction set and in turn improves sensitivity for novel splice junctions and enhance alignment accuracy.

Acceptance thresholds for key quality metrices and mapping parameters along with recommendations for corrective actions when these criteria are not met are provided in the table below:

Parameter	Recommended threshold	Rationale and Corrective actions
Median Phred Per-base quality	Q30	Phred score Q30 denotes 0.1% error probability. Including bases below Q30 is not catastrophic but it includes noisy data into analysis and may impact alignment scores.
Minimum read length after trimming	50 bp	Dropping the cutoff includes shorter reads into alignment steps and may have negative impact in terms of ambiguous mapping, inflated multi-mapping reads and false positives in gene expression.
Adapter content after trimming	0-5%	Lower adapter content is better, but small leftover fractions are common. Higher adapter contents have negative implications as adapter does not match genome, cause mismatches at 3' end and lower the overall mapping quality.
Duplication rates	10-30%	RNA-seq naturally has **higher duplication** due to highly expressed genes. Duplicates should NOT be removed. However, >30% duplication is concerning and there is a trade-off of accepting the data with caveats or may need to redesign the library preparation.
Mapping rate	80-90%	Typically, >80% mapping rate is acceptable, >85% is good and >90% is excellent. For low mapping rates, primary checks should be verifying the appropriate genome and annotations, verifying data quality before/after trimming and finally checking for library contamination using tools like FastQ-screen.

Step 5: Infer data strandedness: day 3

Timing: up to 30 minutes per sample

running infer_experiment.py script:

Determining if data is stranded or un-stranded (dependent of library preparation and sequencing protocols) is crucial for proper analysis and interpretation of RNAseq data. Stranded protocol preserves the directionality of the transcripts, while non-stranded protocol does not.

```
cd ~/RNAseq_Analysis/
cd mapping
```

```
samtools view -b -q 255 control1.Aligned.sortedByCoord.out.bam > c

infer_experiment.py \
-i control1.Aligned.unique.bam \
-s 1000000 \
-r  ~/RNAseq_Analysis/annotations/hg38_GENCODE_V47.bed > control1.

# Repeat above steps for each sample
```

> **Note:** infer_experiment.py performs random sampling of mapped reads and counts the sense cs. antisense strand reads that overlap with annotated genes. The output provides the fraction of reads assigned to each strand, which helps to determine the strandedness of the data. We used uniquely mapped reads (mapping quality score of 255) for this analysis and remove bias towards repetitive regions and multi-mapped reads. The specific parameter `-s 1000000` was used to sample 1 million reads for the inference. This is a common practice to sampling noise in low-depth sequencing data and avoids unreliable strandedness estimates.

accurate inference of strandedness. Using all mapped reads may introduce noise and bias in the inference, especially if there are a significant number of multi-mapped reads.

Data strandedness for each sample was determined using the 'infer_experiment.py' script from the RSeQC package. The output denotes the fraction of reads assigned to each biological sequence orientation (5'-3' – forward or 3'-5' reverse). Generally, for un-stranded libraries, fractions of reads assigned to each orientation are roughly equal (50:50) while for stranded libraries a definitive bias is observed towards one orientation (80:20, 20:80 or similar).

Example output from "infer_experiment.py" script for un-stranded, forward and reverse orientation, respectively.

```
 General output guidelines:
Unstranded -> -s 0 in featureCounts
 Fraction of reads explained by "1++,1--,2+-,2-+": 0.4903
 Fraction of reads explained by "1+-,1-+,2++,2--": 0.4925

Fwd stranded -> -s 1 in featureCounts
 Fraction of reads explained by "1++,1--,2+-,2-+": 0.9441
 Fraction of reads explained by "1+-,1-+,2++,2--": 0.0487

Rev stranded -> -s 2 in featureCounts
 Fraction of reads explained by "1++,1--,2+-,2-+": 0.0441
 Fraction of reads explained by "1+-,1-+,2++,2--": 0.9487
```

Interpretation for "infer_experiment.py" script output

Sequencing Protocol	Library Type	Infer Experiment biased strand	Interpretation	featureCounts option for parameter (-s)
Paired-End	Unstranded	No bias	Unstranded data with roughly equal reads assigned (50:50) on both strands	0
Paired-End	Forward Stranded	1++,1–,2+-,2-+	Forward stranded data with bias towards forward strand (1++,1- -,2+-,2-+)	1
Paired-End	Reverse Stranded	1+-,1-+,2++,2–	Reverse stranded data with bias towards reverse strand (1+-,1-+,2++,2- -)	2

Step 6: Quantification to generate counts matrix: day 3

Timing: up to 30 minutes per sample

Counts matrix in RNAseq:

a. Counts matrix in RNAseq summarizes the expression level by genes in each sample. It is generated by counting the number of reads aligned to each gene.

b. A ‘featureCounts’ program from subread package was employed to count the reads assigned genes in each sample. This program takes genome annotation (GTF format), aligned reads (BAM files from step 2) and inferred strand (strand information from step 3) as inputs, reads are counted and aggregated by each gene, for each sample. From featureCounts’ output column1 and column 7 that correspond to “Gene ID” and “assigned read counts”, respectively were extracted using cut command.

```
############### QUANTIFICATION_PARAMETER S#########
# -T -> number of processors
# -a -> provide annotation file here
# -o -> provide out prefix
# -t -> provide feature type to quantify for
# -g -> attribute to assign the quantification for
# -s -> specify strandedness here, 0 = unstranded, 1 = stranded ar
# -p -> specify is data is paired end
# -B -> quantify if both ends of the pairs are mapped
###############################################

cd ~/RNAseq_Analysis/

mkdir counts
cd counts

featureCounts -T 30 \
-a ~/RNAseq_Analysis/annotations/genome_ref.gtf \
-o control1.ct \
-s 0 \
```

```
-p -B  \
-t gene -g gene_id \
~/RNAseq_Analysis/mapping/control1.Aligned.sortedByCoord.out.bam

# output file "control1.ct" has 7 columns and
# we are interested in column 1 (containing geneID) and column 7 (
# We use `cut` command in Linux to extract only  column 1 and colu
# and save it to another file

grep -v "#" control1.ct | cut -f1,7- > control1.ct.counts

# Repeat above steps for each sample
```

combine counts:

c. Lastly, a custom script was employed to generate a combined counts matrix where each row represents a gene, and each column represents a sample. The values in the matrix denote the number of reads mapped to each gene in each sample.

```
cd ~/RNAseq_Analysis/
cd counts

csvtk join -t $(ls *.counts) | \
sed 's/.Aligned.sortedByCoord.out.bam//g' | \
sed 's/Geneid/Gene_ID/g' > combined_counts.tsv

# Above commands should generate the counts matrix for all the sam
```

Transcripts Per Million (TPM) counts:

d. Transcripts Per Million (TPM) are normalized counts in RNA-seq data. TPM represents the relative abundance of transcripts, essentially indicating the number of reads detected for a gene if sequenced to one million reads. TPM normalizes for both sequencing depth and transcript length, making it useful for comparing gene expression across different samples.

```
cd ~/RNAseq_Analysis/
mkdir TPM
cd TPM
```

```
TPMCalculator -p -e -a -b \
~/RNAseq_Analysis/mapping/control1.Aligned.sortedByCoord.out.bam \
-g ~/RNAseq_Analysis/annotations/genome_ref.gtf

# output file "control1.Aligned.sortedByCoord.out_genes.out"
# will be created containing TPM counts

# extract columns of interest (geneID and TPMcounts)

cut -f 1,7 control1.Aligned.sortedByCoord.out_genes.out > control1

# Repeat above steps for each sample
# combine *.counts files with csvtk

csvtk join -t $(ls *.counts)  > TPM_counts.tsv
```

TPM counts matrix generation:

e. TPMCalculator program add suffix as "#1", "#2", ..., "#N" when the same gene ID is denoted at different locations in annotation (GTF) file. For the sake of simplicity, we keep the gene location with maximum assigned counts so that we retain maximum assigned counts for a specific gene to retain the dominant (highly expressed) transcript. This approach prioritizes the dominant expressed locus for the specific gene while reducing the noise from low-expression transcript or ambiguity of annotated gene models. This approach avoids artificial inflation of counts that may occur if counts are summed across multiple loci. This strategy is opted in the context of the goal gene-level differential expression analysis. For other goals such as isoform specific analyses, current strategy may not be appropriate, and it is recommended to keep and review the counts for duplicated genes.

```
TPM = read.table(file = "TPM_counts.tsv", header = T, sep = "\t",

TPM_nohash = TPM %>%
    dplyr::filter(!str_detect(Gene_ID, "#"))

TPM_withhash = TPM %>%
    dplyr::filter(str_detect(Gene_ID, "#"))
```

```
TPM_withhash$Gene_ID = str_replace(TPM_withhash$Gene_ID, "#.*", "'


TPM_withhash_unique = as.data.frame(TPM_withhash %>%
    rowwise() %>%
    mutate(Total_counts = sum(c_across(2:length(TPM_withhash)))) %
    dplyr::arrange(Gene_ID, desc(Total_counts)) %>%
    dplyr::distinct(Gene_ID, .keep_all = T) %>%
    dplyr::select(-c("Total_counts")))

TPM_final = rbind(TPM_nohash, TPM_withhash_unique)

saveRDS(TPM_final, "TPM_final.rds")
```

Determine correlation between replicates:

The ENCODE recommendations for replicates concordance is - spearman correlation of > 0.9 between isogenic replicates and >0.8 between anisogenic replicates (i.e. replicates from different donors) (Figure 3). We determined the Spearman's correlation among replicates and see high concordance (> 0.9) among replicates.

```
countfile = "counts_Supinoxin.TXT" # provide the path for counts f

raw.data = read.table(countfile, row.names="Gene_ID", header = T,
                      sep = "\t", as.is = T, check.names = F, quot

cor.result <- cor(as.matrix(raw.data), method = "spearman")

corrplot(cor.result,
         method = "circle",
         type = "upper",
         addCoef.col = "white", # Add correlation coefficients as
         number.cex = 0.9, # Adjust size of numbers
         tl.col = "black", # Color of text labels
         tl.srt = 45, # Rotate text labels for better readability
         diag = FALSE
         )
```

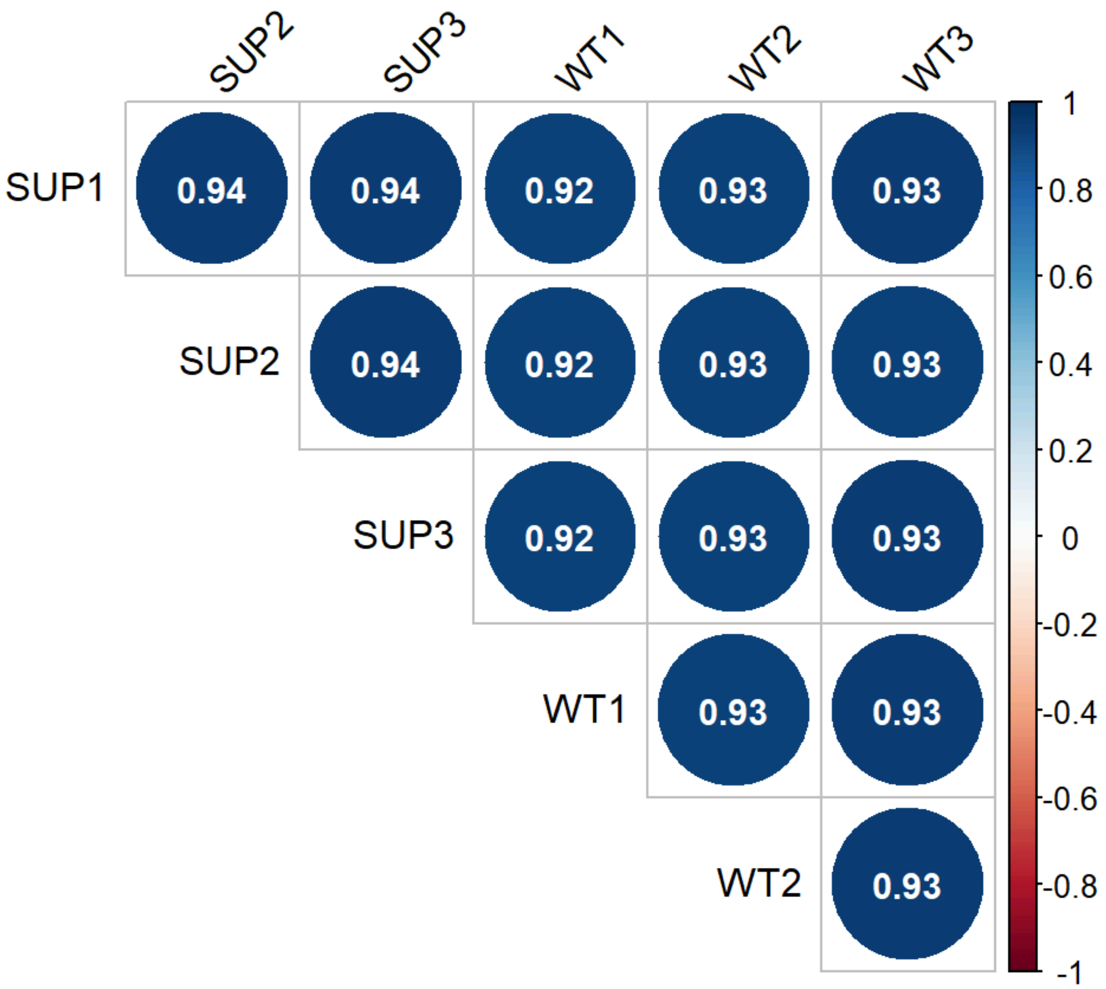

Recommendations for low correlation among replicates:

Correlation thresholds serve as a screening tool and not an automatic exclusion criterion. Low correlation may represent a true biological variation, and it is important to evaluate various technical aspects. For example, one replicate showing low correlation compared to all others (single outlier) may denote sample-specific technical issues and concerned sample should be assessed in terms of sequencing-depth, quality matrices (base-quality, read-length), mapping rate, duplication rate etc. Technical failures like low-depth sequencing, elevated duplication or modest deviation from quality metrices may be addressed by additional sequencing. On the contrary, technical issues such as significantly low mapping rates (<60%), library size < 2x cohort median, duplication rate > 2x cohort median, inconsistent strandedness indicate severe failures and may need assessment for experimental anomalies (e.g. RNA degradation, library preparation artifacts or

contamination) and may flag sample for exclusion. If sequencing was performed in batches, then assessment of batch-effect and batch-correction may be necessary as described elsewhere[SU6.1]. ENCODE recommendation is three replicates for each biological condition[S.

Step 7: Differential Expression (DE) analysis: day 4

Timing: up to 4 hours

DE_edger.R script for differential gene expression:

a. DE analysis identifies genes with significant changes in expression levels between two or more conditions. The analysis involves statistical tests to determine if observed differences in gene expression are likely due to biological factors rather than random noise.

```
# A complete script is available on GitHub as DE_edger.R

library(DESeq2)
library(ggplot2)
library(gplots)
library(tidyverse)
library(RColorBrewer)
library(edgeR)
library(ggrepel)
library(ComplexHeatmap)
library(dplyr)

args <- commandArgs(trailingOnly = TRUE)

countfile = args[1]  #path for the counts file
control = args[2]  #name for control (as in counts file)
treatment = args[3]  #name for treatment (as in counts file)
control_rep = args[4]  #number of replicates for control
treatment_rep = args[5]  #number of replicates for treatment
path = args[6]  #path for annotation file

outprefix <- paste(treatment, control, sep = "_vs_")
```

```
dir.create(outprefix)
setwd(outprefix)

header = paste(rep("#", 50), collapse = "")

sink(file = paste0(outprefix, ".sessioninfo.txt"))

cat(paste(header, "#Version Information", header, sep = "\n"))
cat("\n")
version
cat("\n")

cat(paste(header, "#Session Information", header, sep = "\n"))
cat("\n")
sessionInfo()
sink()

# Save log to file

sink(file = paste0(outprefix, ".log.txt"))

Anno <- read.table(path, sep = "\t", stringsAsFactors = F, header
    quote = "")

raw.data = read.table(countfile, row.names = "Gene_ID", header = T
    sep = "\t", as.is = T, check.names = F, quote = "")

control
treatment

group <- factor(rep(c(control, treatment), times = c(control_rep,
    treatment_rep)), levels = c(control, treatment))

group
control_mat <- select(raw.data, starts_with(control))
treatment_mat <- select(raw.data, starts_with(treatment))
raw.counts <- merge(control_mat, treatment_mat, by = "row.names",
    all.x = TRUE)

raw.counts <- column_to_rownames(raw.counts, var = "Row.names")

#### edgeR Analysis

y <- DGEList(counts = as.matrix(raw.counts), group = group)
x <- calcNormFactors(y)
```

```
keep <- filterByExpr(y)
filtered.data <- y[keep, keep.lib.sizes = FALSE]
y <- calcNormFactors(filtered.data)

design <- model.matrix(~0 + group)

colnames(design) <- levels(y$samples$group)
y <- estimateDisp(filtered.data, design)
fit <- glmQLFit(y, design)
qlf <- glmQLFTest(fit, contrast = c(-1, 1))

edgeR_DE = as.data.frame(topTags(qlf, sort.by = "PValue", n = Inf
edgeR_DE = rownames_to_column(edgeR_DE, "Gene_ID")
edgeR_DE = left_join(edgeR_DE, Anno, by = "Gene_ID")

write.table(edgeR_DE, file = paste0(outprefix, ".DE_edgeR_All.txt
    sep = "\t", quote = F, row.names = F)

saveRDS(edgeR_DE, file = paste0(outprefix, "edgeR_DE.rds"))

#### Filtering EdgeR - FDR 5% filtered
edgeR_DE_FDR5P = dplyr::filter(edgeR_DE, FDR < 0.05)
dim(edgeR_DE_FDR5P)

write.table(edgeR_DE_FDR5P, file = paste0(outprefix, ".DE_edgeR_5
    sep = "\t", quote = F, row.names = F)

sink()
```

b. DE analysis between (DDX5-knockdown and control) and (Supinoxin treated and control) was performed using edgeR R-package.

```
# DE analysis require (1) DE_edger.R (2) count.matrix (3)
# Annotation file <TAB delimited>

# The script was run on windows computer using rstudio as
# below: Please update complete paths for each file

# DE analysis for Supinoxin data:
system("RScript   DE_edger.R   counts_Supinoxin.TXT   H69ARWT   H
```

```
# DE analysis for DDX5KD data:
system("RScript   DE_edger.R   counts_DDX5KD.TXT   WT   DDX5KD  3
```

Step 8: Determine shared up- and down-regulated genes between DDX5KD and Supinoxin data: day 4

Timing: up to 1 hour

Up- and down-regulated genes:

a. In each data, up- and down-regulated genes were denoted as following:
   i. Up-regulated genes - FDR < 0.05 and log2fold-change > 1.
   ii. Down-regulated genes - FDR < 0.05 and log2fold-change < -1.

```
library(dplyr)
library(openxlsx)
library(ggvenn)

setwd("Z:/PCCR/Tran_Elizabeth/Methods_paper/GitHub/data")

SUP.DE = openxlsx::read.xlsx(xlsxFile = "Supinoxin.DE.xlsx",
    sheet = "EdgeR_All")

DDX5KD.DE = openxlsx::read.xlsx(xlsxFile = "DDX5KD.DE.xlsx",
    sheet = "EdgeR_All")

SUP.up = SUP.DE %>%
    dplyr::filter(padj < 0.05) %>%
    dplyr::filter(log2FoldChange > 1)

SUP.down = SUP.DE %>%
    dplyr::filter(padj < 0.05) %>%
    dplyr::filter(log2FoldChange < -1)


DDX5KD.up = DDX5KD.DE %>%
    dplyr::filter(FDR < 0.05) %>%
    dplyr::filter(logFC > 1)
```

```
DDX5KD.down = DDX5KD.DE %>%
    dplyr::filter(FDR < 0.05) %>%
    dplyr::filter(logFC < -1)
```

Venn diagram **(Figure 1A)** for up- and down-regulated genes:

b. A Venn diagram was created using the up- and down-regulated genes from each data using the ggvenn R-package.

```
my_list = list(DDX5KD.up = DDX5KD.up$Gene_ID, DDX5KD.down = DDX5KD
    SUP.down = SUP.down$Gene_ID, SUP.up = SUP.up$Gene_ID)

ggvenn(my_list, show_percentage = F, text_size = 8)
```

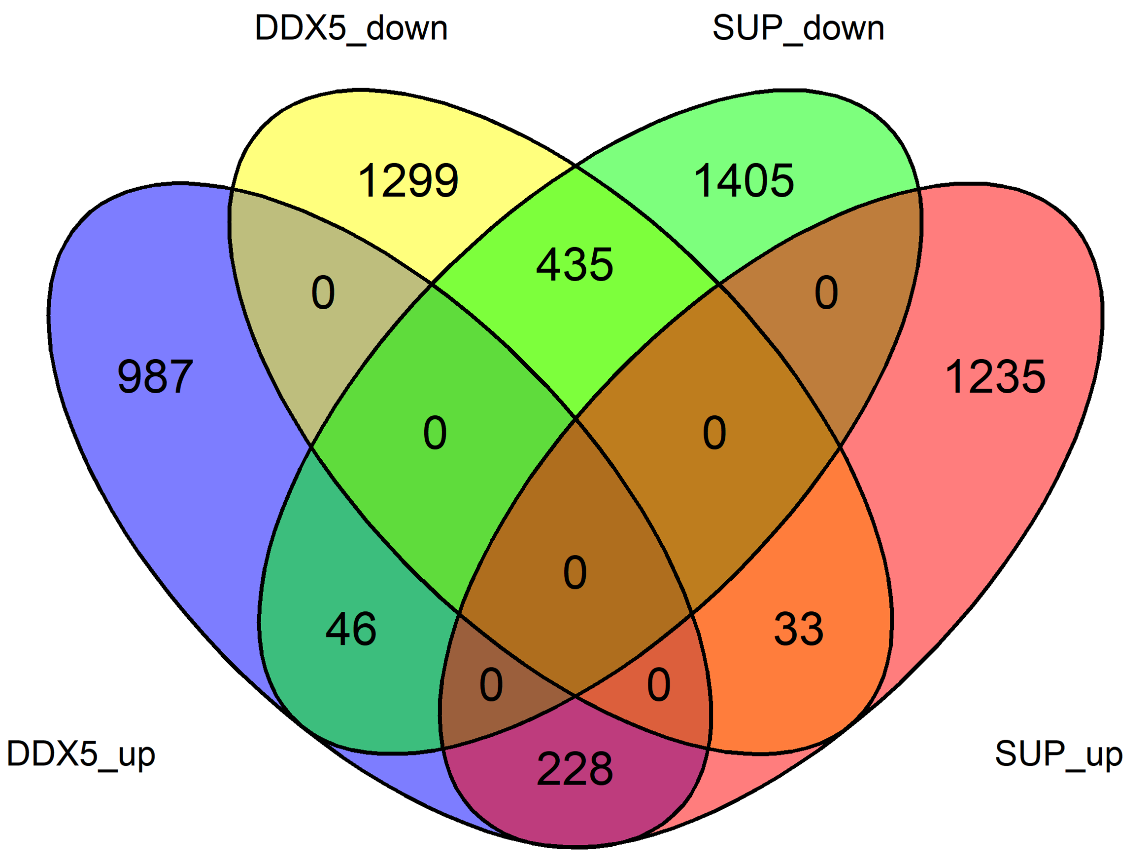

Step 9: Custom figures: day 4

Timing: 2 hours

Heatmap **(Figure 1B)** with individual replicates:

a. Heatmap denoting the expression patterns for key genes among Supinoxin treated and untreated samples was created using R-package ComplexHeatmap.

```
SUP_TPM = readRDS(file = "SUP_TPM.rds")

Anno = read.table("Annotation.TXT", sep = "\t", header = T, quote

Anno_select = Anno %>%
    dplyr::select(all_of(c("Gene_ID", "Gene.name")))

TPM_with_symbol = SUP_TPM %>%
    dplyr::left_join(Anno_select) %>%
    dplyr::select(-c("Gene_ID")) %>%
    dplyr::relocate("Gene.name")
```

```
heat_genes = read.table(file = "heatmap.genes.txt", sep = "\t",
    header = T, quote = "")

mat = TPM_with_symbol %>%
    dplyr::filter(Gene.name %in% heat_genes$symbol) %>%
    remove_rownames() %>%
    column_to_rownames("Gene.name") %>%
    dplyr::relocate(starts_with(c("H69ARSUP"))) %>%
    dplyr::relocate(starts_with(c("H69ARWT")))

heat <- t(scale(t(mat)))

colnames(heat) = c("Untreated 1", "Untreated 2", "Untreated 3",
    "Treated 1", "Treated 2", "Treated 3")

HM1 = ComplexHeatmap::Heatmap(heat, name = "Z-score", width = ncol
    unit(16, "pt"), height = nrow(mat) * unit(10, "pt"), show_row_
    show_column_dend = F, show_row_dend = F, row_names_gp = gpar(f
    column_title = "Heatmap", cluster_columns = F, cluster_rows =
    column_split = c("A", "A", "A", "B", "B", "B"))

# Extract row names
x = ComplexHeatmap::row_order(HM1)
y = rownames(heat)[x]
```

```
y = as.data.frame(y)
colnames(y) = c("Gene_ID")

write.table(as.data.frame(y), file = "heatmap_row_order.txt",
    sep = "\t", quote = F, row.names = F)
```

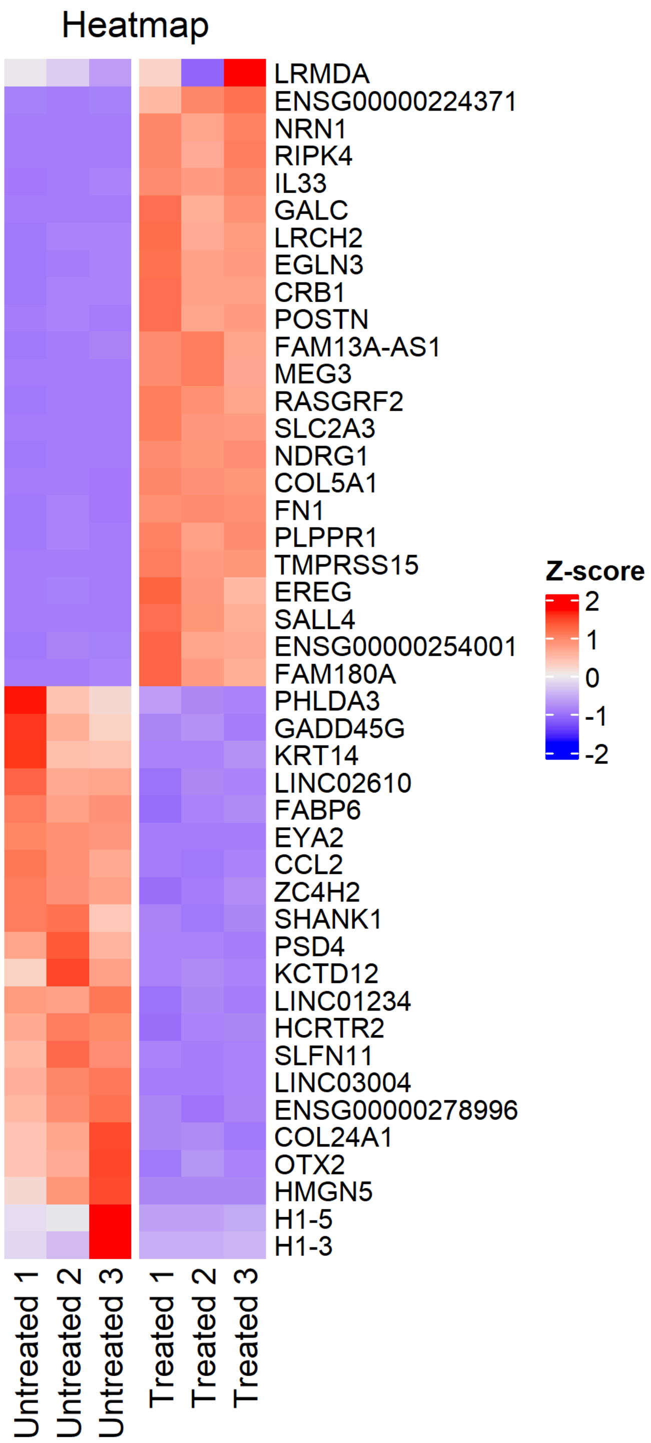

Heatmap **(Figure 1B)** with log2FoldChnage data:

b. Heatmap denoting the up- or down-regulation (log2 fold change) for key genes in Supinoxin treated samples as compared to untreated samples was created using R-package ComplexHeatmap.

```
SUP.DE = openxlsx::read.xlsx(xlsxFile =  "Supinoxin.DE.xlsx", shee

gene_order = read.table(file = "heatmap_row_order.txt", sep = "\t'

heat_data = gene_order %>%
            dplyr::left_join(SUP.DE, by = c("Gene_ID" = "symbol"))
            dplyr::select(all_of(c("Gene_ID","log2FoldChange"))) %
            column_to_rownames("Gene_ID") %>%
            dplyr::rename("LogFC" = "log2FoldChange")

ComplexHeatmap::Heatmap(heat_data,
                        name = "Log 2 FC",
                        width = ncol(heat_data)*unit(40, "pt"),
                        height = nrow(heat_data)*unit(10, "pt"),
                        show_row_names = T,
                        show_column_dend = F,
                        show_row_dend = F,
                        row_names_gp = gpar(fontsize = 10),
                        column_title = "LogFC heatmap",
                        cluster_columns  = F,
                        cluster_rows = F,
                        column_labels = c("Log 2 FC"),
                        cell_fun = function(j, i, x, y, width, hei
                          {
                          grid.text(sprintf("%.1f", heat_data[i, j
                                    x, y, gp = gpar(fontsize = 8))
                        }

)
```

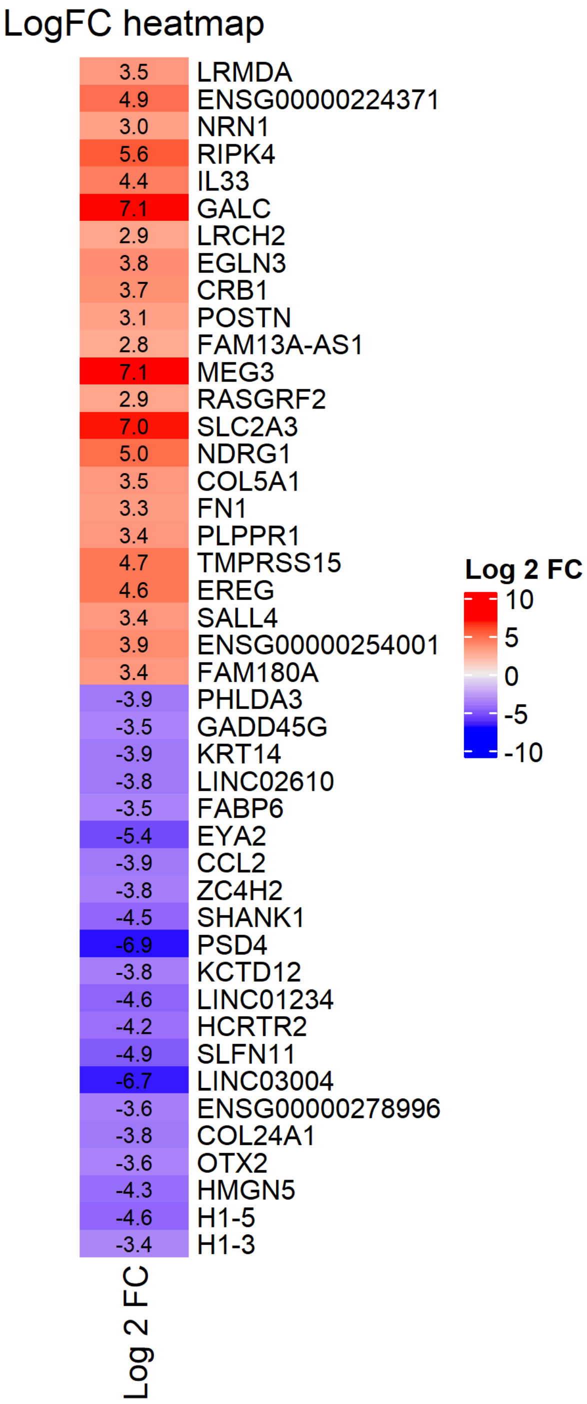

Heatmap **(Figure 1B)** with Average Expression data:

c. Heatmap denoting the average expression across Supinoxin treated and untreated samples was created using R-package ComplexHeatmap.

```
Anno = read.table("Annotation.TXT", sep = "\t", header = T, quote
```

```
gene_order = read.table(file = "heatmap_row_order.txt", sep = "\t'

my_counts = read.table(file = "counts_Supinoxin.TXT", sep = "\t",

my_counts = my_counts %>%
            dplyr::left_join(Anno) %>%
            dplyr::mutate(Gene.name = ifelse(is.na(Gene.name), Ger
            dplyr::select(all_of(c("Gene.name",
                                   "H69ARWT1", "H69ARWT2", "H69ARV
                                   "H69ARSUP1", "H69ARSUP2", "H69A

heat_counts = gene_order %>%
              dplyr::left_join(my_counts, by = c("Gene_ID" = "Gene
              column_to_rownames("Gene_ID") %>%
              dplyr::mutate(AveExpr = rowSums(.)/6) %>%
              dplyr::select(all_of(c("AveExpr")))

col_fun = colorRamp2(c(0, 400), c( "white", "red"))

ComplexHeatmap::Heatmap(heat_counts,
                        name = "AveExpr",
                        col = col_fun,
                        width = ncol(heat_counts)*unit(40, "pt"),
                        height = nrow(heat_counts)*unit(10, "pt"),
                        show_row_names = T,
                        show_column_dend = F,
                        show_row_dend = F,
                        row_names_gp = gpar(fontsize = 10),
                        column_title = "AveExpr heatmap",
                        cluster_columns  = F,
                        cluster_rows = F,
                        cell_fun = function(j, i, x, y, width, hei
                          {
                          grid.text(sprintf("%.0f", heat_counts[i,
                                    x, y, gp = gpar(fontsize = 8))
                        }

)
```

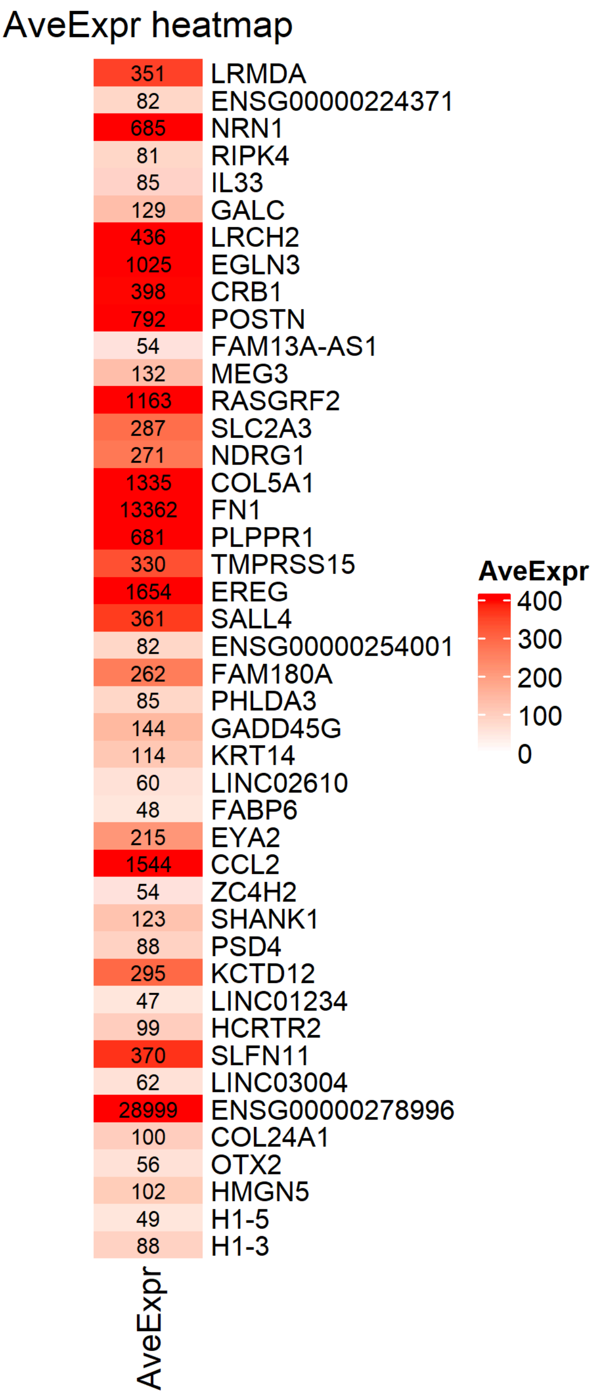

Volcanoplot **(Figure 1C)**:

d. A volcano plot displaying key differentially expressed genes (-Log10PValue on Y-axis) along with up- or down-regulation (log2 fold change on X-axis) in Supinoxin data was created using R-package EnhancedVolcano.

```
SUP.DE = openxlsx::read.xlsx(xlsxFile = "Supinoxin.DE.xlsx",
    sheet = "EdgeR_All")
```

```
label_data = c("EREG", "SLFN11", "CCL2", "EYA2", "PSD4", "LINC0300
    "SDHAF3", "NDUFA1", "COX5B", "UQCRH", "MDH1B", "ACTB", "DDX5",
    "GALC", "MEG3", "SLC2A3", "FN1", "POSTN", "TMPRSS15", "EGLN3",
    "VEGFA", "EREG")

EnhancedVolcano(SUP.DE, lab = SUP.DE$symbol, subtitle = NULL,
    selectLab = label_data, x = "log2FoldChange", y = "pvalue",
    ylab = bquote(~-Log[10] ~ italic(PValue)), title = NULL,
    legendLabels = c("Non-significant", as.expression(bquote("Pass
        Log[2] ~ "fold change cutoff")), "Passed only PValue cutof
        as.expression(bquote("Passed" ~ Log[2] ~ "fold change and
    FCcutoff = 1, pCutoff = 0.05, legendPosition = "right", maxove
    drawConnectors = T)
```

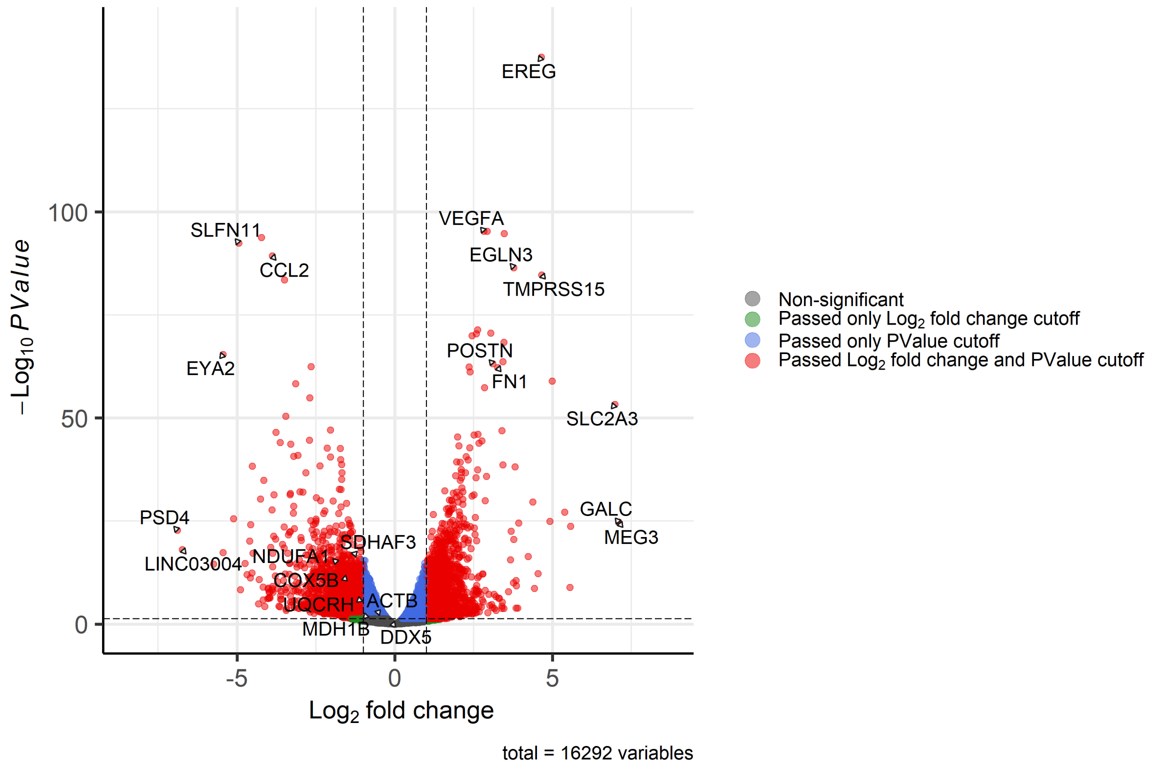

Step 10: Pathway analysis: day 4

Timing: up to 2 hours

Database for pathway analysis:

a. Pathway analysis can be performed using various databases such as KEGG, Reactome, Gene Ontology (GO) and others. The choice of databases depends on the research question and the specific pathways of interest. For this analysis, we started with Hallmark

gene sets from Molecular Signature Database (MSigDB) via R-package msigdbr. The KEGG OXIDATIVE PHOSPHORYLATION (which is the most relevant pathway aligned with current hypothesis) was top enriched pathway in Hallmark gene set.

```
# Pathway analysis (GSEA) for Supinoxin data:

library(clusterProfiler)
library(msigdbr)
library(org.Hs.eg.db)
library(dplyr)

db = "org.Hs.eg.db"

# read “Hallmark” gene set from Molecular Signature
# Database (MSigDB)

hallmark_t2g <- msigdbr(species = "Homo sapiens", category = "H")
    dplyr::select(gs_name, entrez_gene)

SUP_ranked = read.table(file = "SUP_GSEA.rnk", quote = "", sep = '
    header = T)

SUP_ranked = dplyr::arrange(SUP_ranked, desc(Rank))

#######################################

x <- bitr(SUP_ranked$Gene_ID, fromType = "ENSEMBL", toType = "ENTR
    OrgDb = db)

x = x %>%
    dplyr::left_join(SUP_ranked, by = c(ENSEMBL = "Gene_ID")) %>%
    dplyr::select(all_of(c("ENTREZID", "Rank"))) %>%
    dplyr::arrange(desc(Rank))

SUP_genelist = x$Rank
names(SUP_genelist) = x$ENTREZID

#######################################

SUP_GSEA <- GSEA(SUP_genelist, TERM2GENE = hallmark_t2g, , pvalue(

saveRDS(SUP_GSEA, file = "SUP_GSEA_Hallmark.rds")
```

```
write.table(SUP_GSEA, file = "SUP_GSEA_Hallmark_Results.txt",
    sep = "\t", quote = F, row.names = F)
```

b. To dive deeper into pathway mechanisms and canonical signaling, we did second-pass analysis with “C2” curated gene set from MSigDB. Enrichment analysis was performed with pre-ranked genes (Rank = signed fold change * -log10pvalue).

```
# Pathaway analysis (GSEA) for Supinoxin data:

library(clusterProfiler)
library(msigdbr)
library(org.Hs.eg.db)
library(dplyr)

db = "org.Hs.eg.db"

# read “C2” curated gene set from Molecular Signature
# Database (MSigDB)

C2_t2g <- msigdbr(species = "Homo sapiens", category = "C2") %>%
    dplyr::select(gs_name, entrez_gene)
```

> **Note:** A different MSigDB database of choice can be updated in the function above to perform enrichment with different database.

Enrichment analysis for Supinoxin data:

c. Enrichment analysis for Supinoxin data was performed using the GSEA function available in clusterProfiler R-package.

```
SUP_ranked = read.table(file = "SUP_GSEA.rnk", quote = "", sep = '
    header = T)

SUP_ranked = dplyr::arrange(SUP_ranked, desc(Rank))

#######################################

x <- bitr(SUP_ranked$Gene_ID, fromType = "ENSEMBL", toType = "ENTR
    OrgDb = db)
```

```
x = x %>%
    dplyr::left_join(SUP_ranked, by = c(ENSEMBL = "Gene_ID")) %>%
    dplyr::select(all_of(c("ENTREZID", "Rank"))) %>%
    dplyr::arrange(desc(Rank))

SUP_genelist = x$Rank
names(SUP_genelist) = x$ENTREZID

#######################################

SUP_GSEA <- GSEA(SUP_genelist, TERM2GENE = C2_t2g, , pvalueCutoff

saveRDS(SUP_GSEA, file = "SUP_GSEA.rds")

write.table(SUP_GSEA, file = "SUP_GSEA_Results.txt", sep = "\t",
    quote = F, row.names = F)
```

Supinoxin barplot **(Figure 2A)**:

d. A barplot for important enriched pathways in Supinoxin data was created using GSEA results and ggplo2 R-package.

```
SUP_GSEA = read.table(file = "SUP_GSEA_Results.txt", header = T,
    sep = "\t", quote = "")

my_pathways = c("KEGG_RIBOSOME", "KEGG_OXIDATIVE_PHOSPHORYLATION",
    "KEGG_VALINE_LEUCINE_AND_ISOLEUCINE_DEGRADATION", "KEGG_PROTEA
    "KEGG_DNA_REPLICATION", "KEGG_PYRIMIDINE_METABOLISM", "KEGG_GL
    "KEGG_TRYPTOPHAN_METABOLISM", "KEGG_PEROXISOME", "KEGG_PURINE_

SUP_GSEA_select = SUP_GSEA %>%
    dplyr::filter(ID %in% my_pathways) %>%
    dplyr::select(all_of(c("ID", "pvalue"))) %>%
    dplyr::mutate(log.Pvalue = log10(pvalue) * -1)

SUP_GSEA_select$ID = str_replace_all(SUP_GSEA_select$ID, "KEGG_",
    "")
SUP_GSEA_select$ID = str_replace_all(SUP_GSEA_select$ID, "_",
    " ")
SUP_GSEA_select$ID = str_to_sentence(SUP_GSEA_select$ID)

SUP_GSEA_select$ID = str_replace_all(SUP_GSEA_select$ID, "Dna ",
    "DNA ")
```

```
ggplot(data = SUP_GSEA_select, aes(x = reorder(ID, log.Pvalue),
    y = log.Pvalue)) + ylab(bquote(~-Log[10] ~ italic(PValue))) +
    xlab("Enriched Pathways") + geom_bar(colour = "black", fill =
    stat = "identity", width = 0.5) + coord_flip() + theme(axis.te
    colour = "black")) + theme(axis.title = element_text(size = 7,
    face = "bold")) + theme(panel.background = element_rect(fill =
    theme(axis.line = element_line(colour = "black", linewidth = 0
    theme(plot.title = element_text(color = "black", size = 8,
        face = "bold", hjust = 0.5)) + ggtitle("Enriched pathways
    geom_hline(yintercept = 1.3, linetype = "dashed")
```

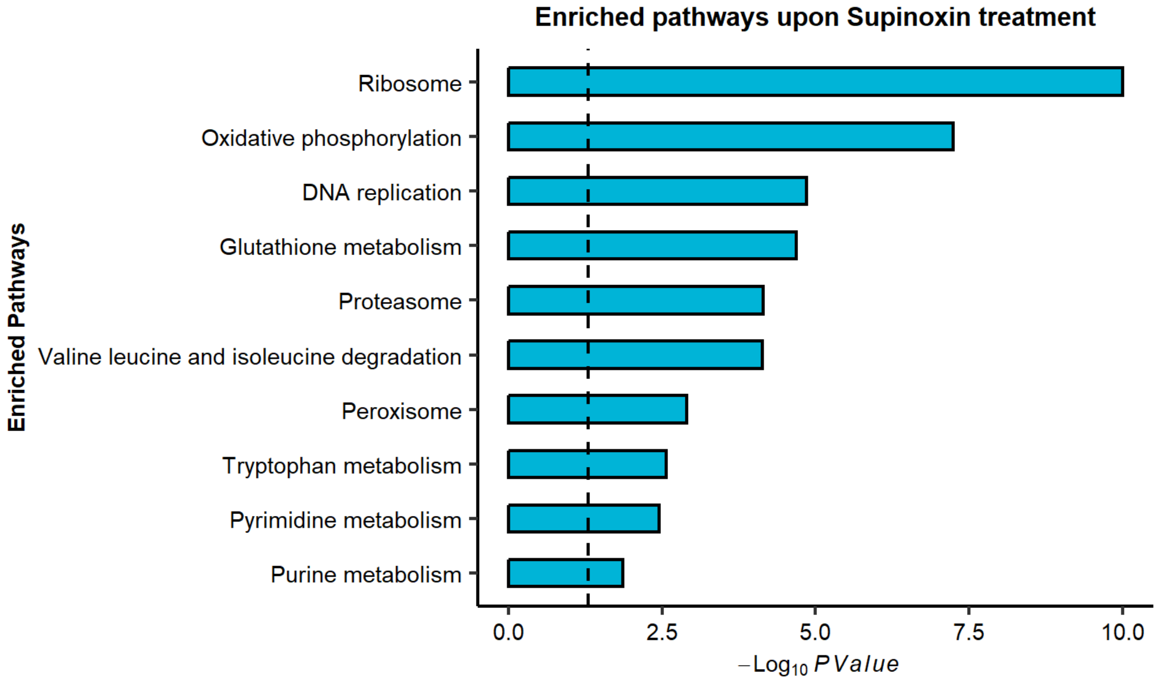

Enrichment analysis for DDX5 knockdown data:

e. Enrichment analysis for DDX5 knockdown data was performed using the GSEA function available in clusterProfiler R-package.

```
# Pathaway analysis (GSEA) for DDX5KD data:

library(clusterProfiler)
library(msigdbr)
library(org.Hs.eg.db)
library(dplyr)

db = "org.Hs.eg.db"

# read “C2” curated gene set from Molecular Signature
# Database (MSigDB)
```

```
C2_t2g <- msigdbr(species = "Homo sapiens", category = "C2") %>%
    dplyr::select(gs_name, entrez_gene)

DDK5KD_ranked = read.table(file = "DDK5KD_GSEA.rnk", quote = "",
    sep = "\t", header = T)

DDK5KD_ranked = dplyr::arrange(DDK5KD_ranked, desc(Rank))

x <- bitr(DDK5KD_ranked$Gene_ID, fromType = "ENSEMBL", toType = "E
    OrgDb = db)

x = x %>%
    dplyr::left_join(DDK5KD_ranked, by = c(ENSEMBL = "Gene_ID")) %
    dplyr::select(all_of(c("ENTREZID", "Rank"))) %>%
    dplyr::arrange(desc(Rank))


DDK5KD_genelist = x$Rank
names(DDK5KD_genelist) = x$ENTREZID


DDK5KD_GSEA <- GSEA(DDK5KD_genelist, TERM2GENE = C2_t2g, pvalueCut

write.table(DDK5KD_GSEA, file = "DDK5KD_GSEA_Results.txt", sep = '
    quote = F, row.names = F)
```

compareCluster **(Figure 2B)**:

f. The compareCluster function from clusterProfiler R-package was applied to examine biological (reference: C2 database from MSigDB) profiles of Supinoxin and DDX5 knockdown data and subsequent dot plot displaying simultaneous enrichment of important pathways in each data.

```
C2_t2g <- msigdbr(species = "Homo sapiens", category = "C2") %>%
    dplyr::select(gs_name, entrez_gene)

SUP_ranked = read.table(file = "SUP_GSEA.rnk", quote = "", sep = '
    header = T)

SUP_ranked = dplyr::arrange(SUP_ranked, desc(Rank))

x <- bitr(SUP_ranked$Gene_ID, fromType = "ENSEMBL", toType = "ENTF
```

```
    OrgDb = db)

x = x %>%
    dplyr::left_join(SUP_ranked, by = c(ENSEMBL = "Gene_ID")) %>%
    dplyr::select(all_of(c("ENTREZID", "Rank"))) %>%
    dplyr::arrange(desc(Rank))

SUP_genelist = x$Rank
names(SUP_genelist) = x$ENTREZID

##################################

DDK5KD_ranked = read.table(file = "DDK5KD_GSEA.rnk", quote = "",
    sep = "\t", header = T)

DDK5KD_ranked = dplyr::arrange(DDK5KD_ranked, desc(Rank))

x <- bitr(DDK5KD_ranked$Gene_ID, fromType = "ENSEMBL", toType = "E
    OrgDb = db)

x = x %>%
    dplyr::left_join(DDK5KD_ranked, by = c(ENSEMBL = "Gene_ID")) %
    dplyr::select(all_of(c("ENTREZID", "Rank"))) %>%
    dplyr::arrange(desc(Rank))

DDK5KD_genelist = x$Rank
names(DDK5KD_genelist) = x$ENTREZID

##################################

my_list = list(DDX5KD = DDK5KD_genelist, Supinoxin = SUP_genelist)

CCS = compareCluster(my_list, fun = "GSEA", TERM2GENE = C2_t2g,
    pvalueCutoff = 1)

colnames(CCS@compareClusterResult)[7] = "PValue"
CCS@compareClusterResult$ID = str_replace_all(CCS@compareClusterRe
    "KEGG_", "")
CCS@compareClusterResult$Description = str_replace_all(CCS@compare
    "KEGG_", "")
CCS@compareClusterResult$Description = str_to_sentence((CCS@compar

my_pathways = c("RIBOSOME", "OXIDATIVE_PHOSPHORYLATION", "PROTEASO
    "DRUG_METABOLISM_CYTOCHROME_P450", "TRYPTOPHAN_METABOLISM")
```

```
my_pathways = str_to_sentence(my_pathways)


x = CCS@compareClusterResult
x$row.ID = rownames(x)

select.pathways = x %>%
    dplyr::filter(Description %in% my_pathways) %>%
    dplyr::pull("Description")

enrichplot::dotplot(CCS, showCategory = select.pathways, color = '
```

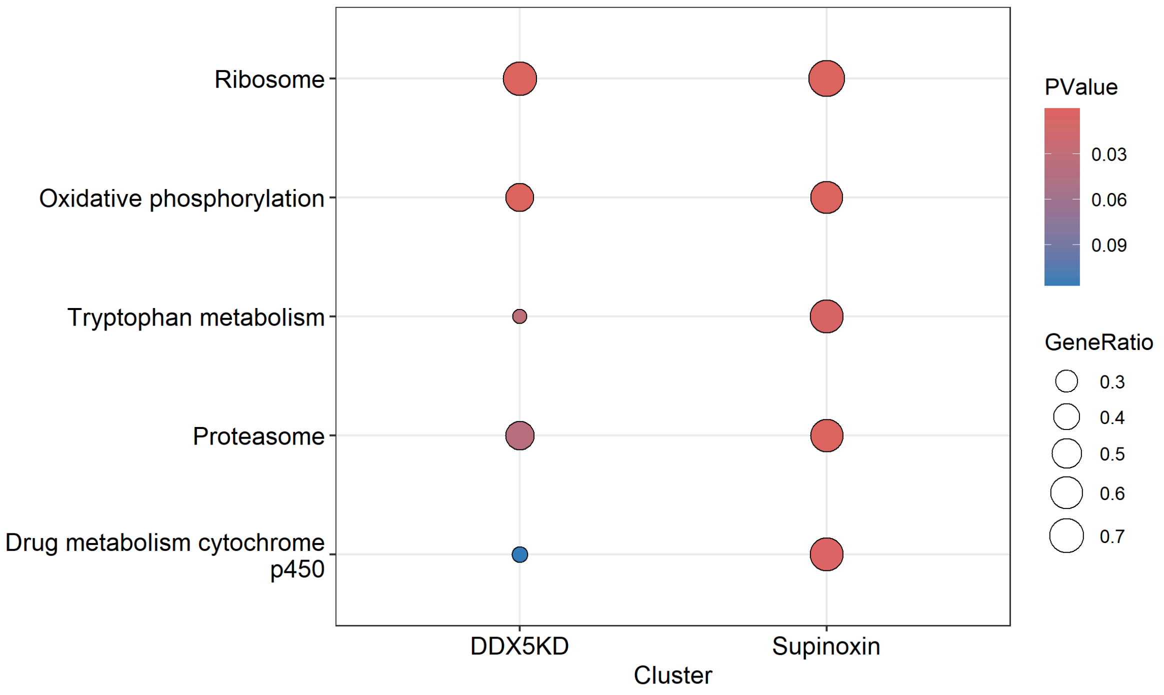

Supinoxin gseaplot **(Figure 2C)**:

g. The gseaplot was created to visualize the distribution of the gene set and the enrichment score for KEGG_OXIDATIVE_PHOSPHORYLATION pathway in Supinoxin data.

```
SUP_GSEA = readRDS(file = "SUP_GSEA.rds")

my.index = which(SUP_GSEA@result$Description == "KEGG_OXIDATIVE_PH

gseaplot(SUP_GSEA, geneSetID = my.index, by = "runningScore",
    title = SUP_GSEA$Description[my.index])
```

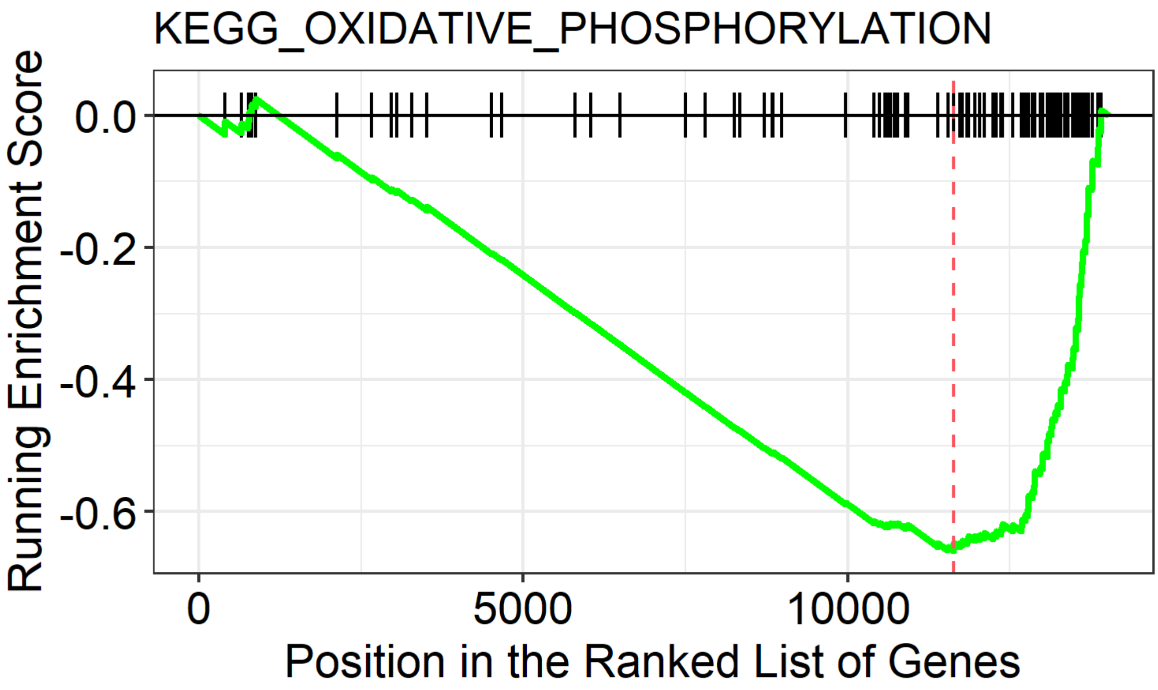

Supinoxin cnetplot **(Figure 2D)**:

h. Gene-concept network plotfor pathway “KEGG OXIDATIVE PHOSPHORYLATION” in Supinoxin data was created using the cnetplot function from clusterProfiler R-package.

```
SUP_ranked = read.table(file = "SUP_GSEA.rnk", quote = "", sep = '
    header = T)

SUP_ranked = dplyr::arrange(SUP_ranked, desc(Rank))

x <- bitr(SUP_ranked$Gene_ID, fromType = "ENSEMBL", toType = "ENTR
    OrgDb = db)

SUP.DE = openxlsx::read.xlsx(xlsxFile = "Supinoxin.DE.xlsx",
    sheet = "EdgeR_All")

x = x %>%
    dplyr::left_join(SUP.DE, by = c(ENSEMBL = "Gene_ID")) %>%
    dplyr::select(all_of(c("ENTREZID", "log2FoldChange")))

logFC_genelist = x$log2FoldChange
names(logFC_genelist) = x$ENTREZID

SUP_GSEA = setReadable(SUP_GSEA, "org.Hs.eg.db", "ENTREZID")

p = cnetplot(SUP_GSEA, showCategory = SUP_GSEA@result$Description[
    foldChange = logFC_genelist, categorySize = "pvalue")
```

```
min.value = -2
max.value = 2

p + scale_colour_gradient2(name = "fold change", low = "blue",
    mid = "white", high = "red", limits = c(min.value, max.value),
    breaks = c(min.value, 0, max.value))
```

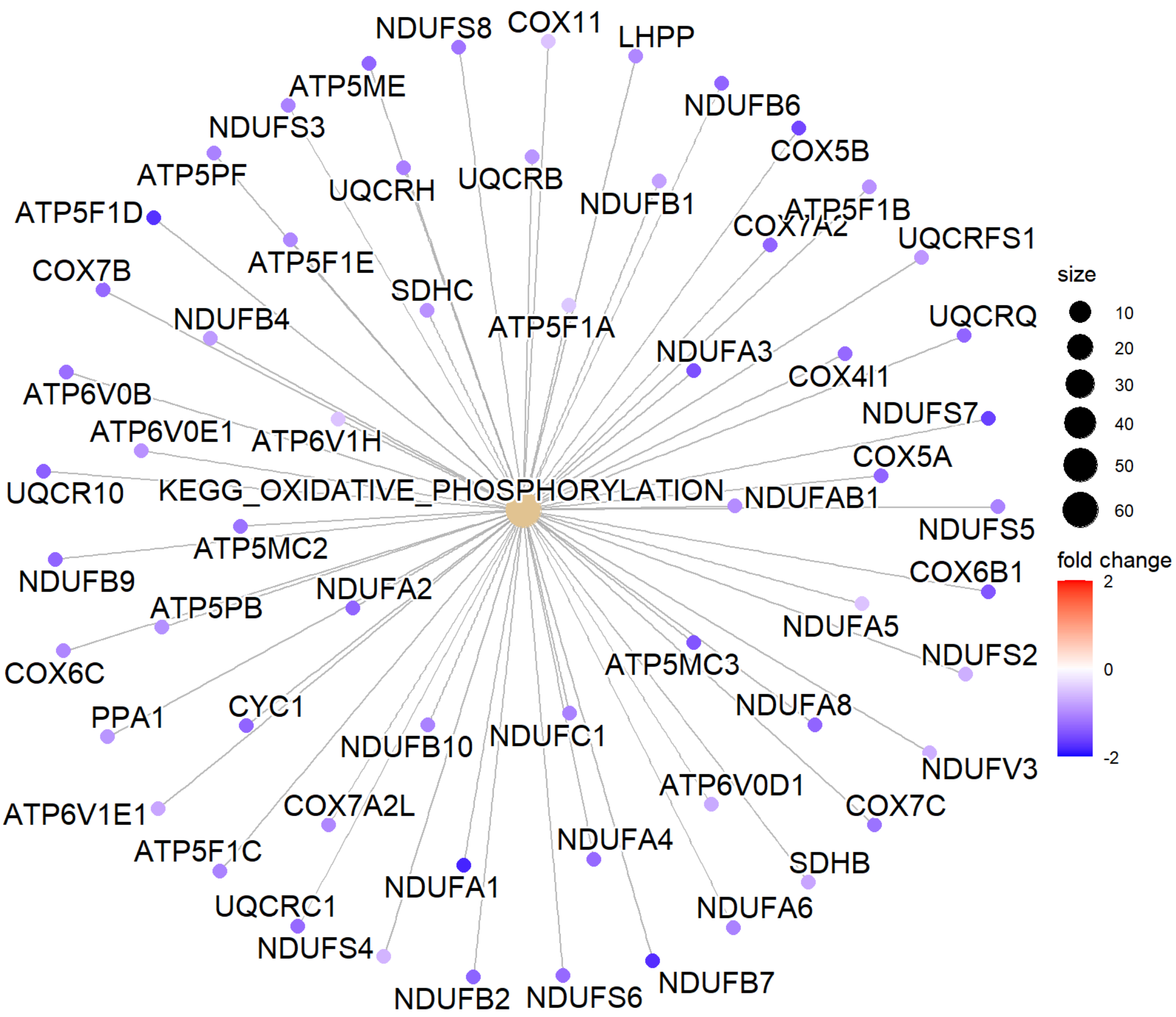

Step 11: Confidence Assessment for KEGG Oxidative Phosphorylation (OXPHOS) Network:

Load STRING Database:

To assess the robustness of gene-concept network within the KEGG Oxidative Phosphorylation (OXPHOS) pathway, we determined if transcriptional enrichment (*adjusted pvalue* = 3.90E-06, normalized

enrichment score = -2.11) correspond to subsequent protein-level organization.

```
# Load Required Libraries
# STRINGdb      : Access STRING protein-protein interactions
# clusterProfiler: GSEA results handling
# igraph        : Network construction and topology metrics
# org.Hs.eg.db  : Gene ID conversion (ENTREZ ↔ SYMBOL)


library(STRINGdb)
library(clusterProfiler)
library(igraph)
library(org.Hs.eg.db)

db = "org.Hs.eg.db"

# Initialize STRING Database
# version 11.5
# species 9606 (human)
# score_threshold = 700 (high-confidence interactions)

string_db <- STRINGdb$new(
  version = "11.5",
  species = 9606,
  score_threshold = 700
)

# Load Precomputed Results
# gsea_result : GSEA enrichment results
# deg         : Differential expression results (edgeR output)

gsea_result = readRDS("SUP_GSEA.rds")
deg = readRDS("SUP_DE.rds")

# Extract OXPHOS Pathway from GSEA Results

gsea_df <- as.data.frame(gsea_result)

# Filter specifically for KEGG Oxidative Phosphorylation pathway
oxphos_row <- gsea_df %>%
          dplyr::filter(Description == "KEGG_OXIDATIVE_PHOSPHORYLA

# Compute Pathway-Level Confidence
```

```
# term_confidence integrates:
#   - NES magnitude (biological direction/strength)
#   - Adjusted p-value (statistical significance)

term_confidence <- abs(oxphos_row$NES) * -log10(oxphos_row$p.adjus

# Extract Core Enrichment Genes from GSEA
# core_enrichment contains ENTREZ IDs separated by "/"

core_entrez <- unlist(strsplit(oxphos_row$core_enrichment, "/"))

# Convert ENTREZ IDs to gene symbols for STRING compatibility
core_genes <- bitr(core_entrez,
                      fromType = "ENTREZID",
                      toType   = "SYMBOL",
                      OrgDb    = org.Hs.eg.db)

# Subset Differential Expression Results
# Keep only genes participating in OXPHOS core enrichment

deg_subset <- deg[deg$Gene.name %in% core_genes$SYMBOL, ]

# Compute Gene-Level Confidence
# Combines:
#   - Effect size (|logFC|)
#   - Statistical confidence (-log10 FDR)
# Higher value = strong and statistically reliable gene regulation

deg_subset$gene_confidence <- abs(deg_subset$logFC) * -log10(deg_s

mean_gene_conf <- mean(deg_subset$gene_confidence, na.rm = TRUE)
```

Mapping OXPHOS genes to STRING Database:

Gene symbols from OXPHOS pathway were mapped to corresponding STRING IDs. The majority of genes were successfully mapped and interaction querying yielded greater than 2000 pairwise connections among 60 OXPHOS gene symbols indicating extensive inter-protein connectivity and coherent protein interaction network. Visualization of resulting STRING network demonstrate a densely interconnected structure with highly significant *pvalue*.

```r
# Map Gene Symbols to STRING IDs
# Required for retrieving PPI interactions

mapped_genes <- string_db$map(
  deg_subset,
  "Gene.name",
  removeUnmappedRows = TRUE
)

string_ids <- mapped_genes$STRING_id

# Retrieve All Interactions Between OXPHOS STRING IDs

interactions <- string_db$get_interactions(string_ids)
dim(interactions)  # shows number of interaction pairs retrieved

# Quick visualization of full STRING network
string_db$plot_network(mapped_genes$STRING_id)
```

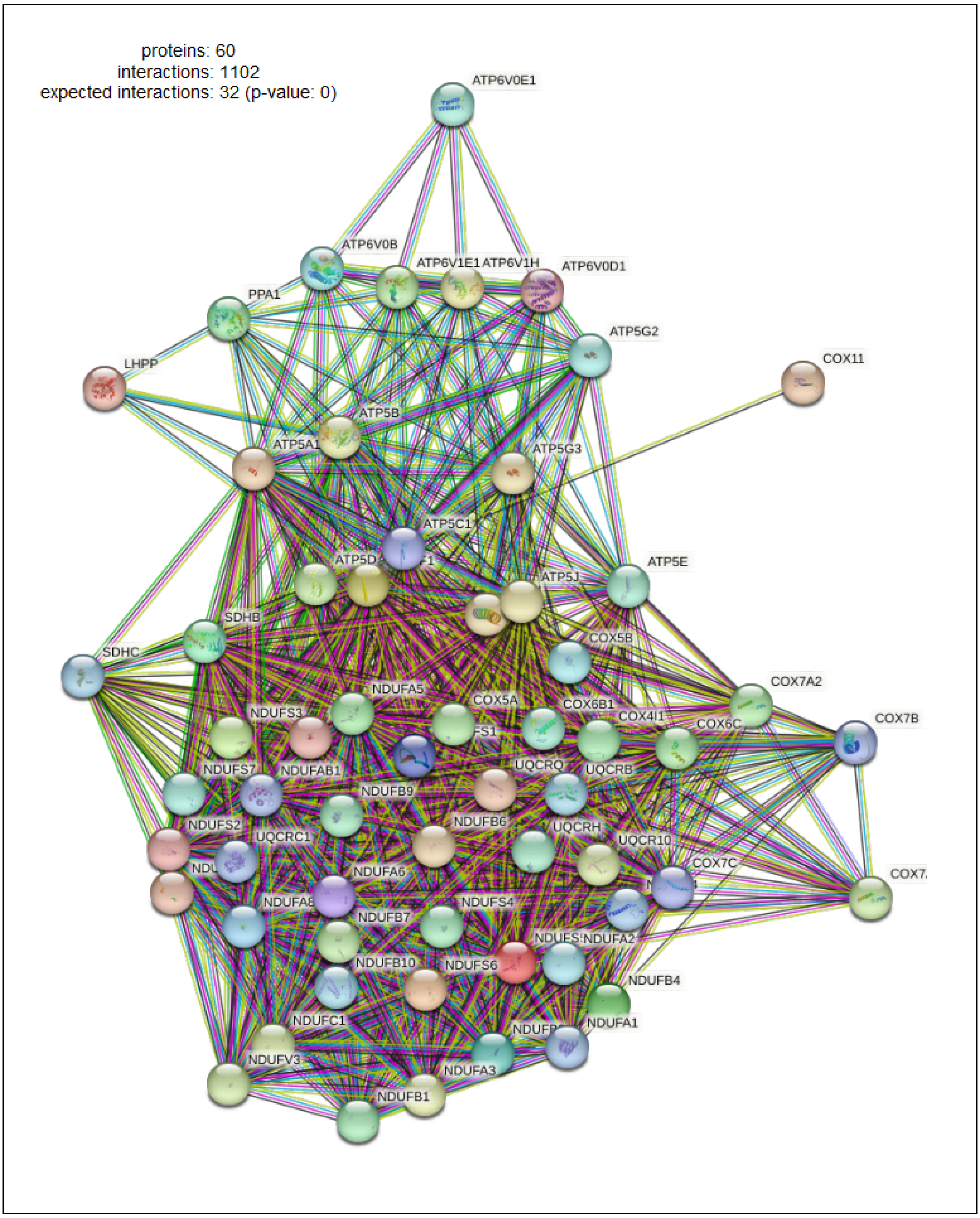

Assessment of OXPHOS protein-protein interactions:

Each STRING interaction within this network is supported by **combined confidence score** metric which is computed by combining the probabilities from the different evidence channels (e.g. experimental data, conserved neighborhood, Gene fusions, Phylogenetic co-occurrence, Co-expression, Database imports). STRING combined confidence score ranges from 0-1000 (some sources may show range from 0-1 which simply divide the scores by 1000). The combined

confidence score is interpreted as (0-400 = low-confidence; 400-700 = high-confidence and 900-1000=very high-confidence). Networks with low-confidence scores generally have more connecting edges, dense network and may contain indirect evidence with higher chances of false positives while networks with high-confidence scores (>700) have fewer edges with strong experimental support and clean core network with higher biological evidence.

```
library(dplyr)
library(ggplot2)

# Examine Distribution of STRING Confidence Scores
# combined_score ranges from 700–1000 (high-confidence threshold a
# This step visualizes robustness of interaction evidence

df_binned <- interactions %>%
  mutate(
    bin = cut(
      combined_score,
      breaks = seq(700, 1000, by = 100),
      include.lowest = TRUE,
      right = FALSE
    )
  ) %>%
  count(bin) %>%
  mutate(percent = n / sum(n) * 100)



ggplot(df_binned, aes(x = bin, y = percent)) +
  geom_col(fill = "#2C7BB6") +
  geom_text(aes(label = sprintf("%.1f%%", percent)),
            vjust = -0.5, size = 5) +
  scale_y_continuous(expand = expansion(mult = c(0, 0.1))) +
  labs(
    x = "Score Interval",
    y = "Percentage of Values",
    title = "Distribution of STRING Confidence Scores"
  ) +
  theme_minimal(base_size = 14)
```

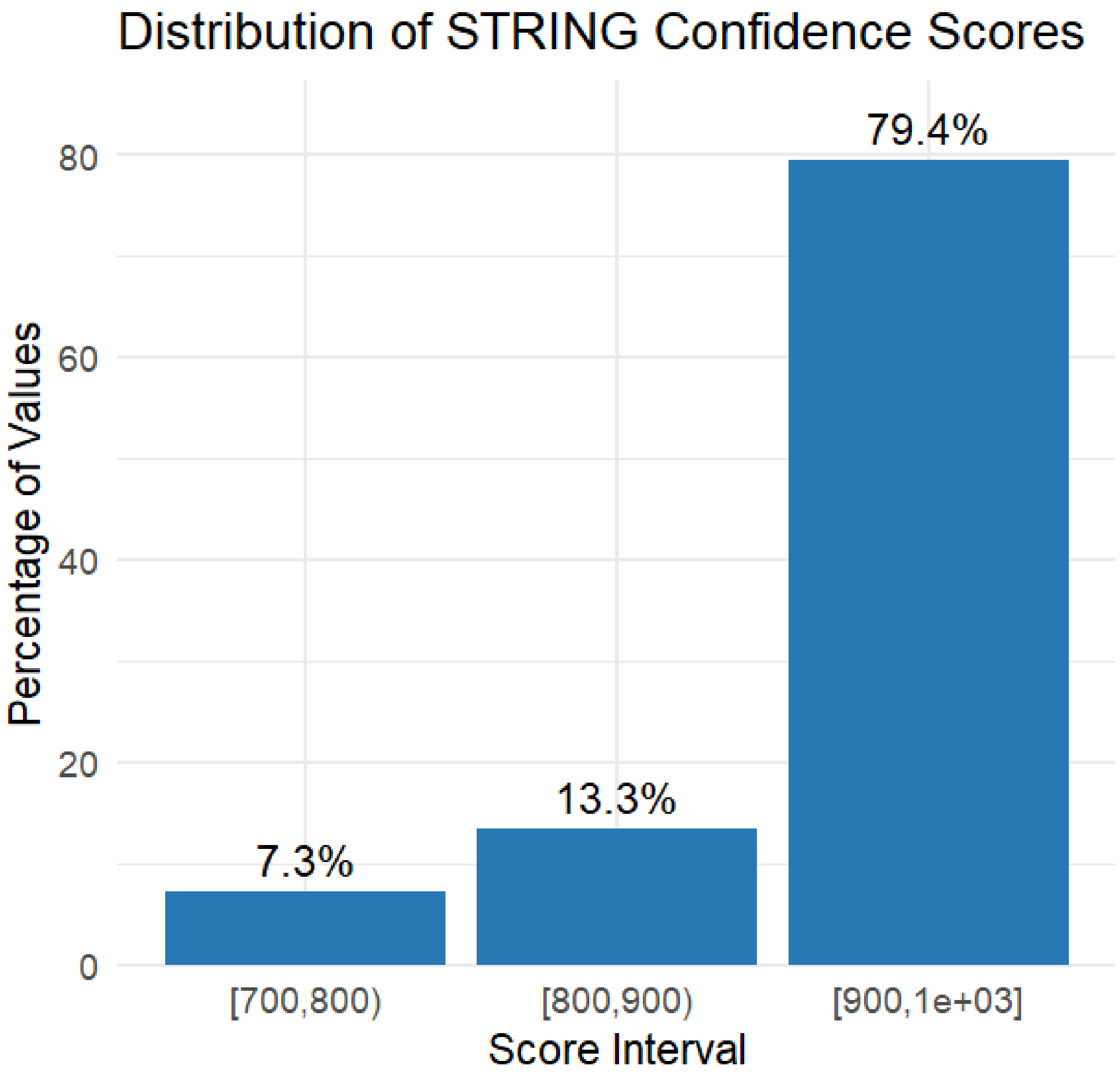

Distribution of STRING confidence scores within OXPHOS interaction network was examined. As denoted in the plot above, >79% interactions within OXPHOS network have very-high-confidence (>900) score and remaining >20% interactions have high-confidence scores (>700) score indicating highly structured and biologically cohesive protein interaction network.

Enrichment analysis of OXPHOS protein-protein interactions:

Next, protein–protein interaction (PPI) enrichment analysis was performed. The OXPHOS network contains 1102 observed edges (ppi_edges) compared to 32 expected edges (ppi_lambda). PPI enrichment analysis *pvalue* (ppi_pvalue) was reported as 0, indicating highly significant overrepresentation of interactions than random chance. To account for machine precision limits (i.e. *pvalue* reported as 0), the smallest positive double value was used to compute a conservative −log10-transformed enrichment metric, yielding a robust

quantitative estimate of PPI confidence. Further, a **composite network confidence score** was computed as function of OXPHOS pathway enrichment significance, average of gene-level differential expression strength and PPI enrichment magnitude. High composite network confidence score affirms the significance of OXPHOS pathway network.

```
# Perform STRING PPI Enrichment Test
# Tests whether observed connectivity >> expected random connectiv

ppi_enrichment <- string_db$get_ppi_enrichment(string_ids)

ppi_pvalue <- ppi_enrichment$enrichment   # enrichment p-value
ppi_edges  <- ppi_enrichment$edges        # observed edges
ppi_lambda <- ppi_enrichment$lambda       # expected edges

# Handle Extremely Small p-value
# If enrichment p = 0 (underflow), substitute smallest positive do

min_p <- .Machine$double.xmin
ppi_confidence <- -log10(min_p)

# Composite Network Confidence Score
# Integrates:
#   - Pathway-level significance
#   - Gene-level differential expression strength
#   - PPI enrichment strength

network_confidence_score <- term_confidence * mean_gene_conf * ppi
```

Topological analysis of OXPHOS protein-protein interactions:

Next, topological analysis was performed to quantitatively characterize the structural organization of a protein–protein interaction (PPI) network. Topological analysis revealed the organization of protein interactions and associated key genes (termed as hub genes) that are strong contributors to network integrity. First an igraph object (g) was built from OXXPHOS interactions followed by simplification of graph object by removing duplicate edges and self-loops (i.e. removing technically redundant interactions). Resulting simplified graph was comprised of 60 nodes and 1,102 non-redundant edges and

corresponding network density of 0.62 (i.e. 62% of all theoretically possible pairwise interactions are retained within simplified graph object). Subsequent calculation of degree centrality identified nodes with high number of connections. Nodes with highest degree centrality were used to determine top 10 hub genes that form core components within the OXPHOS network. Visualization of core OXPHOS network (i.e. protein-protein interactions associated with top 10 hub genes) was performed.

```
# Build igraph Object for Topological Analysis

library(igraph)

g <- graph_from_data_frame(
       interactions[, c("from", "to")],
       directed = FALSE)

# Remove duplicate edges or self-loops for accurate topology
g <- simplify(g)

# Basic Network Metrics
num_nodes   <- vcount(g)
num_edges   <- ecount(g)
density_val <- edge_density(g)

# Degree centrality (number of connections per gene)
deg_cent <- igraph::degree(g)

# Identify Top Hub Genes
# Select top 10 genes with highest connectivity

hub_genes <- names(sort(deg_cent, decreasing = TRUE))[1:10]

# Convert STRING IDs back to gene symbols for interpretation
hub_ids <- mapped_genes$Gene.name[mapped_genes$STRING_id %in% hub_

hub_interactions <- mapped_genes %>%
                       dplyr::filter(Gene.name %in% hub_ids)

# Plot network focusing only on hub genes
string_db$plot_network(hub_interactions)
```

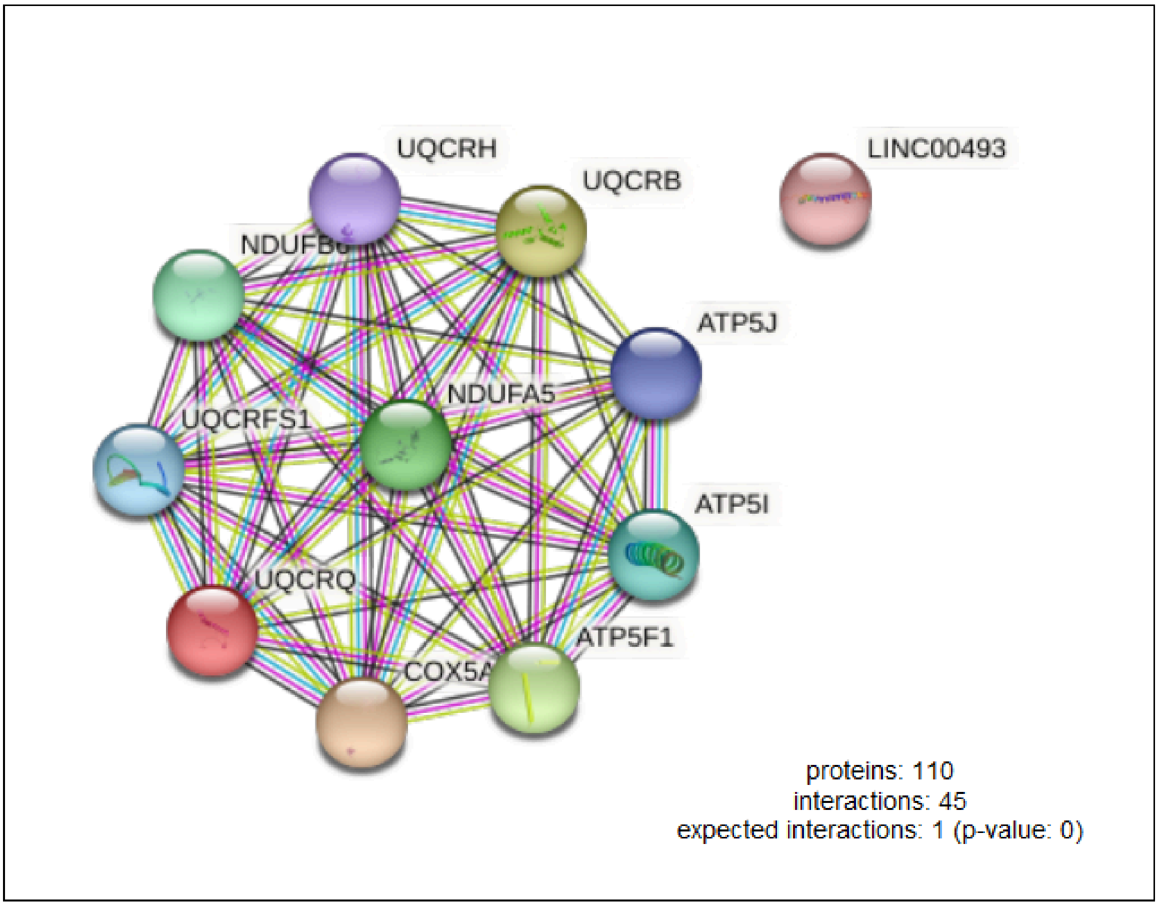

Conclusion:

First, the mapping of OXPHOS network to STRING interactions indicate extensive inter-protein connectivity and >79% interactions with very high confidence score (>900) denote biological and functional relevance of protein-protein interactions. Enrichment testing of protein-protein interactions revealed highly significant *pvalue* confirming that PPI network connectivity exceeds random expectation. Finally, topological analysis moved beyond simple interaction counts and quantitatively characterized the structural organization of a protein–protein interaction network and revealed the hub genes that contribute most strongly to network integrity. Collectively, these analyses indicate that OXPHOS pathway in not only transcriptionally enriched but also forms a structured, biologically cohesive protein-protein interaction network with hierarchical organization and central hub genes. This reinforces our findings that OXPHOS perturbations are coordinated pathway level remodeling activity rather than simple gene-level variations.
